# Stimulus-independent and stimulus-dependent neural networks underpin placebo analgesia responsiveness in humans

**DOI:** 10.1038/s42003-023-04951-7

**Published:** 2023-05-27

**Authors:** Lewis S. Crawford, Noemi Meylakh, Paul M. Macey, Vaughan G. Macefield, Kevin A. Keay, Luke A. Henderson

**Affiliations:** 1 School of Medical Sciences (Neuroscience), Brain and Mind Centre, University of Sydney, Sydney, NSW 2006 Australia; 2 UCLA School of Nursing, University of California, Los Angeles, CA 90095 USA; 3 Department of Neuroscience, Monash University, Melbourne, VIC 3800 Australia

**Keywords:** Sensory processing, Neural circuits

## Abstract

The neural circuits that regulate placebo analgesia responsivity are unknown, although engagement of brainstem pain modulatory regions is likely critical. Here we show in 47 participants that differences are present in neural circuit connectivity’s in placebo responders versus non-responders. We distinguish stimulus-independent and stimulus-dependent neural networks that display altered connections between the hypothalamus, anterior cingulate cortex and midbrain periaqueductal gray matter. This dual regulatory system underpins an individual’s ability to mount placebo analgesia.

## Abstract (web summary)

An fMRI study in 47 participants reveals that stimulus-independent and stimulus-dependent neural networks centered around the midbrain periaqueductal gray underpin an individual’s ability to mount placebo analgesia.

## Introduction

Placebo analgesia is a powerful phenomenon in which an inert substance or visual cue that provokes positive expectations^1^, conditioning effects^2^, or environmental associations^3–5^ evokes pain inhibition. It is thought that placebo analgesia involves the recruitment of descending projections from prefrontal and cingulate cortices to the brainstem pain modulating center, the midbrain periaqueductal gray matter (PAG)^4,6,7^. Since placebo analgesic effects can be reduced by opioid antagonists and stimulation of the ventrolateral column of the PAG (vlPAG) produces opioid-mediated analgesia^5,7^, it has long been thought that the vlPAG is responsible for placebo analgesia. However, a recent ultra-high field functional magnetic resonance imaging (fMRI) study demonstrated that it is the lateral PAG (lPAG), which produces non-opiate mediated analgesia, and not the vlPAG that is critical for placebo analgesia^8^.

Preclinical investigations have revealed that lPAG stimulation evokes emotional coping behaviors, of which analgesia is an integral component^9^. While the lPAG can produce these behaviors without input from higher centers, it was shown over half a century ago that the sensitivity of lPAG is tonically regulated by hypothalamic inputs^10–13^. In humans, the hypothalamus forms part of the lower pain control system and is involved in both pain control and maintaining autonomic homeostasis via its reciprocal connection with the lPAG^14,15^. Additionally, both hypothalamic and midbrain activation has been observed during placebo analgesia, suggesting a phylogenetically conserved system of pain control exists consisting of subcortical and brainstem structures including the hypothalamus and PAG^16^.

Importantly, placebo analgesic responses are not expressed in all individuals and what determines placebo analgesia responsivity remains unknown. Given the abovementioned preclinical and human data, it is possible that on-going modulation of the lPAG by the hypothalamus determines whether or not an individual expresses placebo analgesia. Placebo analgesia is also associated with noxious stimulus-evoked activity changes in higher brain regions including the rostral anterior cingulate cortex (rACC) and dorsolateral prefrontal cortex (dlPFC)^6,7^. These activation patterns are accompanied by heightened µ-opioid binding in the rACC and coupling between the rACC and PAG^17,18^, and the expression of placebo analgesia can be blocked by the administration of the opioid antagonist naloxone. When naloxone is administered, the attenuation of these responses is associated with a reduction in rACC-PAG connectivity^7^.

Given these observations, we hypothesized a two-network model of brain regulation of placebo analgesia. That is, placebo responsivity will depend on lPAG regulation by two distinct networks: i) a *stimulus-independent network* that includes the hypothalamus, and tonically regulates lPAG sensitivity, and ii) a *stimulus-dependent network* that includes the rACC, and phasically alters lPAG activity to produce placebo-mediated reductions in perceived pain intensity.

**Fig. 1:**
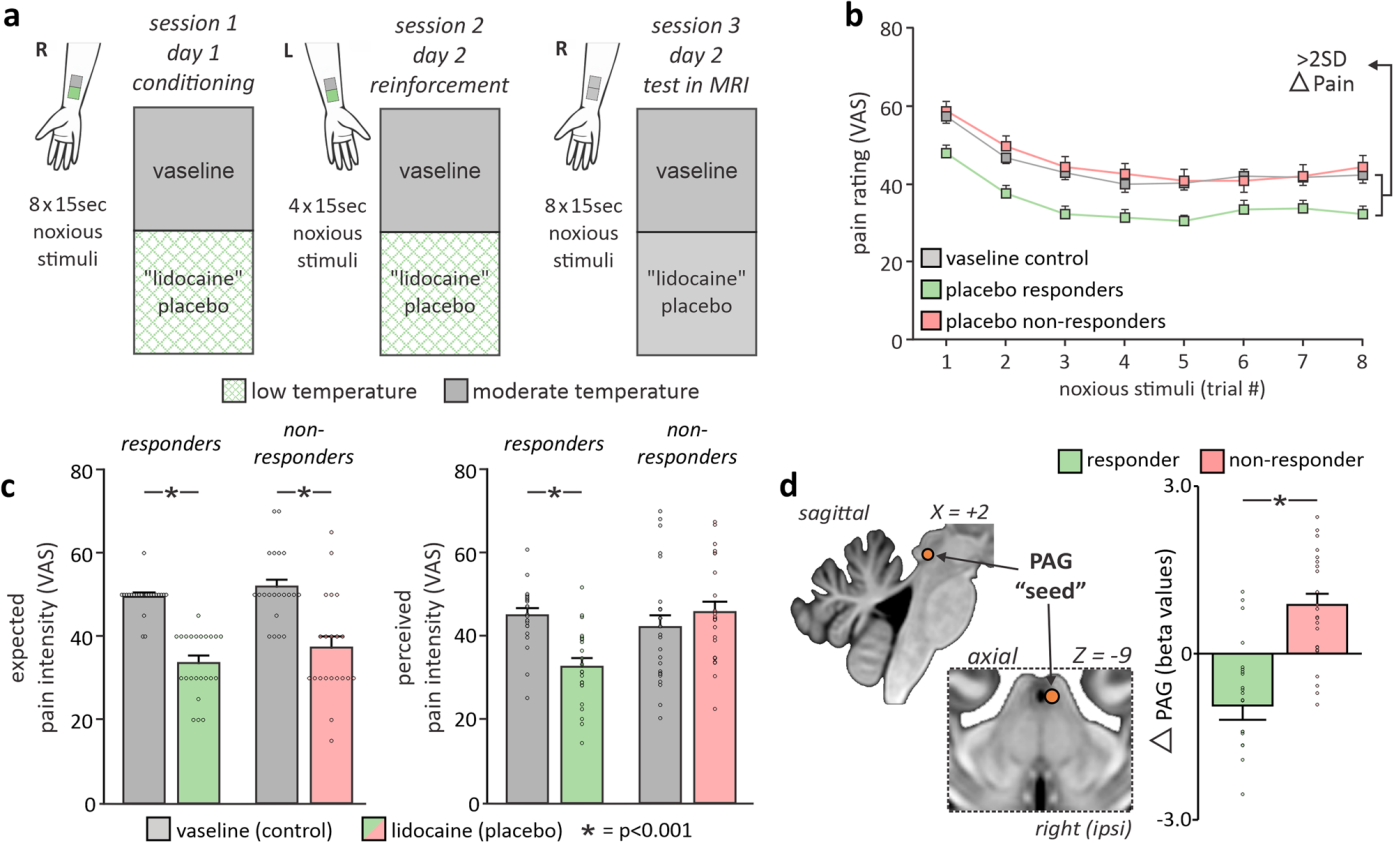
Experimental protocol, placebo-related activity, and connectivity functional maps. **a** Placebo induction. Conditioning was performed by applying low intensity noxious stimuli to the lidocaine-site and moderate intensity to the vaseline-site; crucially, during this phase participants believed stimuli of moderate intensity were being applied to both sites. On the following day, a reinforcement phase was conducted using the low and moderate temperatures on the opposite forearm. Then, after a washout period, two independent functional magnetic resonance imaging (fMRI) series were collected where we applied identical moderate intensity noxious stimuli to the control vaseline (scan 1), and placebo ‘lidocaine’ cream (scan 2) sites sequentially. During these two series, participants rated their expected and perceived pain on an MR-compatible visual analog scale (*0* *=* *no pain, 100* *=* *worst pain imaginable*). **b** Perceived pain intensities during noxious stimuli. Mean (±SEM) pain intensity ratings during the placebo lidocaine-site scan in placebo responder (*n* = 23; green) and non-responder (*n* = 24; pink) groups, relative to the average pain ratings from all 47 participants during the control vaseline-site scan (gray). **c** Expected and perceived pain intensities. The difference in expected and reported pain directly prior and during the two series in placebo responder and non-responder groups. *p < 0.001. **d** Midbrain periaqueductal gray matter (PAG) signal intensity changes. Brainstem maps representing differences in noxious-stimulus evoked signal intensity changes during the placebo lidocaine- and control vaseline-site scans were entered into a 2-sample group analysis which compared placebo responder (*n* = 23) and non-responder (*n* = 24) groups. A significant cluster with a peak within the lateral PAG emerged. Beta values were extracted from a 1 mm diameter sphere at the peak of this cluster and plotted. This sphere was used as the “seed” for subsequent analyses. Box plots depict the mean values for each group and cluster with error bars representing ±SEM surrounding this mean.

By deceptively applying different intensity short-lasting thermal stimuli onto sites on the arm, we conditioned healthy participants to believe a placebo cream (labeled “lidocaine”) was acting to reduce their pain relative to an adjacent control cream (labeled “vaseline”). In a subsequent session, whilst collecting ultra-high-field (7 Tesla), high-resolution (1 × 1 × 1.2 mm voxel) fMRI, we applied identical intensity stimuli to both creams (“vaseline”/control; “lidocaine”/placebo) and recorded subjective pain responses in 47 participants (25 male; mean ± SD age 24.0 ± 3.8) (Fig. 1a). We classified individuals as responder (*n* = 23) or non-responder (*n* = 24) using the two-standard deviation band method^19^ (Fig. 1b), and conducted group-level analyses using SPM12 and custom software to explore changes in signal intensity, stimulus-independent connectivity (functional connectivity), and stimulus-dependent (psychophysiological interaction) connectivity associated with placebo responses. Although it has long been proposed that top-down recruitment of analgesic brainstem pathways underpins placebo analgesia, information on directionality of seed-to-voxel relationships cannot be gleaned from these connectivity analyses alone^20^. As such we additionally conducted Dynamic Causal Modeling (DCM) and a multiple mediation analysis to determine directed connectivity between cortical and subcortical regions (i.e., if placebo analgesia was associated with top-down or bottom-up projections), as well as determine which regions were working either independently or as a system to drive the relationship between lPAG activity and placebo responses.

## Results

### Expectation independent placebo analgesia is associated with altered functional activation in the lateral PAG

Throughout the experiment, participants rated their pain continuously by sliding a cursor connected to a visual analog scale (VAS), extending from 0 (no pain) to 100 (worst pain imaginable). Despite both groups expecting reduced pain on the placebo lidocaine-treated site (*mean* *±* *SEM expectation* responder: vaseline = 49.3 ± 0.8, lidocaine = 33.5 ± 1.6, *p* < 0.001; non-responder: vaseline = 51.7 ± 1.8, lidocaine = 37.1 ± 1.6, *p* < 0.001), only 23 of the 47 participants demonstrated a significant pain reduction when identical intensity stimuli were applied to both sites (*mean* *±* *SEM VAS* responder: vaseline = 45.2 ± 1.5, lidocaine = 32.9 ± 1.9, *p* < 0.001; non-responder: vaseline = 42.2 ± 2.8, lidocaine = 45.9 ± 2.4, *p* = 0.09) (Fig. 1c, Supplementary Fig 1). Pain rating responses to the control vaseline-site did not differ between response and non-responder groups (*F*_2,46_ = 2.59, *p* = 0.22). Additionally, inspection of the low and moderate temperatures applied throughout conditioning and test phases revealed no differences between placebo responder and non-responder groups (Supplementary Table 1). Group-level analyses of placebo responder and non-responder groups revealed a significant and differential engagement of the lPAG, consistent with a previous report (*mean* *±* *SEM change in β value* responder: −0.56 ± 0.33; non-responder: 1.15 ± 0.24; *p* < 0.001) (Fig. 1d). A 1 mm radius sphere at the peak of this cluster was used as a seed region for subsequent connectivity analyses.

### Placebo analgesia relates to ongoing coupling changes between the midbrain PAG and subcortical limbic sites

**Fig. 2:**
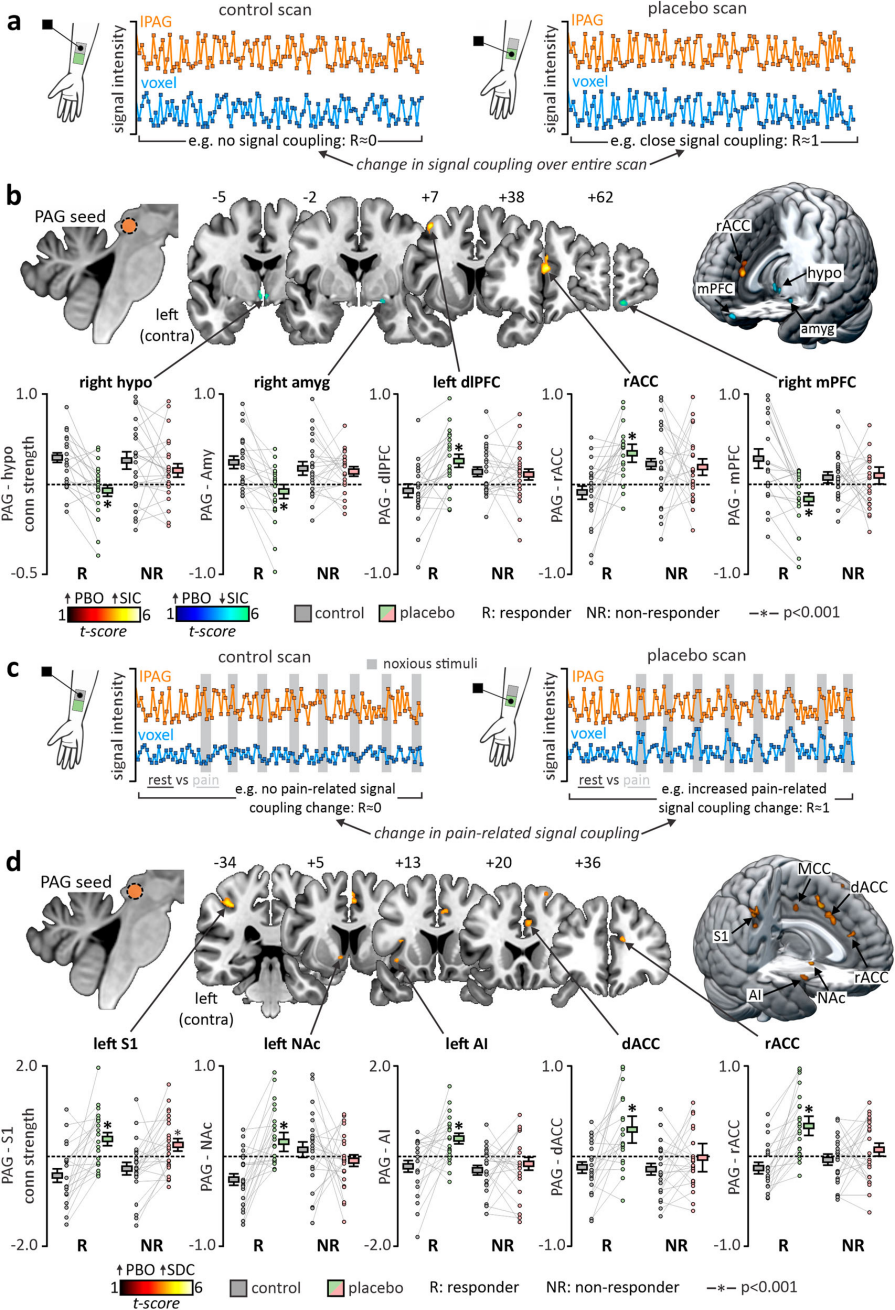
Stimulus-independent and stimulus-dependent cortico-brainstem connectivity changes during placebo analgesia. **a** Functional Connectivity (FC) Analysis. Functional connectivity determines areas which alter in coupling with a seed region across the entire scan. Positive values indicate a correlation between a seed and voxel timeseries, whereas a negative value indicates anticorrelation. Control vaseline- and placebo lidocaine-site functional scans were analyzed, allowing us to determine which brain areas altered their ongoing, stimulus-independent coupling with the lateral midbrain periaqueductal gray matter (lPAG) during placebo analgesia. **b** Voxel-by-voxel FC analysis in placebo responders. Paired analysis (control vaseline versus placebo lidocaine-site scans) in placebo responders (*n* = 23) revealed a pattern of stimulus independent connectivity changes. Relative to the control vaseline-site, connectivity decreased during the stimulation of the placebo lidocaine-site between the lPAG and the left and right posterior hypothalamus (hypo), right medial nucleus of the amygdala (amyg), and right medial prefrontal cortex (mPFC), and increased with both the left dorsolateral prefrontal cortex (dlPFC) and rostral anterior cingulate cortex (rACC). Non-responders (*n* = 24) displayed no significant connectivity changes between the two functional series in these same brain regions. **c** Psychophysiological Interaction (PPI) Analysis. PPI connectivity analysis considers the timeseries of an elected region (seed) activity during time-specific events. During the control vaseline- and placebo lidocaine-site scans, eight noxious stimuli were delivered at 8 time points, allowing us to determine which brain regions altered their connectivity’s in a stimulus-dependent manner. **d** Voxel-by-voxel PPI analysis in placebo responders. Paired analysis (control vaseline- versus placebo lidocaine-site scans) in placebo responders (*n* = 23) revealed significant increases in pain-related connectivity between the lPAG and the contralateral primary somatosensory cortex (S1), anterior insula cortex (AI), nucleus accumbens (NAc), rACC, dorsal ACC (dACC), and mid cingulate cortices (MCC). Non-responders displayed no significant pain-related connectivity changes in all of these brain regions, apart from S1 in which both responders and non-responders displayed connectivity increases during placebo lidocaine- compared with control vaseline-site scans. Box plots depict the mean values and individual participant connectivity data for each group and cluster with error bars representing ±SEM surrounding this mean.

To explore the presence of a *stimulus-independent* network communicating with or receiving information from the lPAG, we conducted a functional connectivity (FC) analysis using the lPAG seed timeseries (Fig. 2a, b; Table 2). A paired, 2nd-level, voxel-by-voxel analysis was conducted using resulting contrast images from the placebo responder group to identify regions which independent of when noxious stimuli were applied, altered their pattern of coherence with the seed timeseries between the stimulation of control vaseline-treated and placebo lidocaine-treated sites. That is, how cortical regions changed in their communication with the lPAG between contexts of pain and placebo.

In support of our hypothesis, we identified a stimulus-independent network consisting largely of limbic subregions in which placebo responders displayed marked decreases in functional connectivity between the stimulation of control-treated and placebo-treated sites. Specifically, we observed reductions in functional connectivity between the bilateral posterior hypothalamus (PH) and the lPAG (Fig. 2b, Table 1). Whilst PH-lPAG coupling was strong during the control-site scan, it was negligible during the placebo-site scan. Similar coupling changes were observed between the lPAG and both the medial nucleus of the amygdala (MeA) and medial prefrontal cortex (mPFC), such that whilst these regions were tightly coupled during typical pain perception, in participants that developed significant placebo analgesic responses, these regions together reduced in their functional coupling with the lPAG. With previous associations between these specific regions and emotional processing during pain and threat responses^17–19^, our results indicate that during contexts of placebo, this circuit alters its contact with descending brainstem pathways to establish an appropriate setting for endogenous pain inhibition.

**Table 1:** Location, level of significance, and cluster size of regions altering in connectivity with the right lateral PAG across the entire scan timecourse in the placebo responder group.

	MNI coordinates			*t*-value	cluster size	PAG Whole scan connectivity change (mean ± SEM)		Responder: nonresponder connectivity
	X	Y	Z			Control scan	Lidocaine scan	Change (*p*-value)
Functional Connectivity (FC)								
PBO > Control								
Ipsilateral rACC	3	40	17	5.10	311	−0.009 ± 0.008	0.035 ± 0.006	0.01
Contralateral dlPFC	−29	25	27	4.16	98	−0.007 ± 0.007	0.025 ± 0.008	0.004
PBO > Control								
Ipsilateral mPFC	11	60	−18	4.58	203	0.029 ± 0.011	−0.016 ± 0.007	0.007
Ipsilateral MeA	21	−2	−13	3.90	44	0.025 ± 0.007	−0.008 ± 0.008	0.006
Ipsilateral Posterior Hypothalamus	3	−5	−8	4.64	40	0.023 ± 0.006	−0.007 ± 0.006	0.04
Contralateral Posterior Hypothalamus	−1	−4	−7	5.63	83	0.020 ± 0.005	−0.012 ± 0.006	0.01

The level of significance between conditions and groups is provided in the right most column. Co-ordinates are in Montreal Neurological Institute (MNI) space. Cluster sizes are derived from resliced 1 mm isotropic image series. “ipsilateral” = right.

*rACC* rostral anterior cingulate cortex, *dlPFC* dorsolateral prefrontal cortex, *mPFC* medial prefrontal cortex, *MeA* medial nucleus of the amygdala.

In addition to decreases in limbic subregion coupling with the lPAG, stimulus-independent connectivity increases were also observed between the lPAG and both the dlPFC and rACC. These two regions displayed largely opposing responses to that previously described such that coupling was negligible during typical pain processing and markedly increased during stimulation of the placebo-treated site. The dlPFC and ACC reciprocally communicate and have been heavily implicated in both placebo responsiveness, as well as higher-order emotional processing during pain^2,20^. Indeed, non-invasive stimulation of the dlPFC can both attenuate and promote placebo analgesia^21,22^, and this finding supports a circuit between the dlPFC, rACC, and PAG as critical in the maintenance of successful placebo responses^23^.

To explore if these pattern of functional connectivity changes were isolated to placebo responders, significant clusters were saved as volume-of-interest (VOI) masks and measures of functional connectivity from each cluster were extracted from the placebo nonresponder group. Indeed, no significant change in functional coupling between any site detailed in the *stimulus independent* network was identified in placebo nonresponders (Supplementary Table 2), suggesting that these group of regions played a functional role in initiating placebo responses via their altered ongoing coupling with the lPAG. We additionally inspected if, within either group, these regions displayed altered activation during either the stimulation of the control- or placebo-treated sites. In neither the placebo responder or nonresponder group did we observe any significant difference in activation change in any of the *stimulus independent network* regions (Supplementary Table 3).

Taken together, these findings suggest that limbic sites such as the PH and MeA, specifically via altered coupling with the lPAG and not necessarily pain-related activation can influence the emergence of a significant placebo response.

### Increased pain-related coupling between the PAG and frontotemporal sites are present during placebo analgesia

By convolving the seed timeseries with scan timepoints where noxious stimuli were applied, a psychophysiological interaction analysis was conducted to determine regions which altered in lPAG coupling specifically during the perception of pain in placebo – i.e. a *stimulus dependent network*. Once again, we conducted a paired analysis using placebo responder contrast images this time revealing cortical sites that altered in coupling with the lPAG specifically between noxious events in the context of pain and placebo (Fig. 2c).

No single region could be identified as reducing in lPAG coupling during placebo relative to pain. However, a collection of regions previously identified as responsible for the pain percept and its modulation displayed increases in noxious-evoked connectivity change with the seed during the placebo-site versus control-site scans. Specifically, placebo responders displayed significant increases in pain-related connectivity between the lPAG and the primary somatosensory cortex (S1), anterior insula (AI), nucleus accumbens (NAc), supplementary motor area (SMA), as well as the rostral (rACC), dorsal (dACC), and mid (MCC) cingulate cortices (Fig. 2d, Table 2).

**Table 2:** Location, level of significance, and cluster size of regions altering in connectivity with the right lateral PAG specifically during stimulus application in the placebo responder group.

	MNI coordinates			*t*-value	Cluster size	PAG stimulus-dependent connectivity change (mean ± SEM)		Responder: nonresponder connectivity
	X	Y	Z			Control scan	Lidocaine scan	Change (*p*-value)
Psychophysiological Interaction (PPI)								
PBO > control								
Contralateral Anterior Insula	−37 −33	12 13	−7 11	3.64 3.65	72 31	−0.26 ± 0.12 −0.23 ± 0.13	0.40 ± 0.14 0.40 ± 0.11	0.03 0.04
Contralateral NAcc	−7	5	−5	4.27	64	−0.26 ± 0.06	0.16 ± 0.10	0.003
Ipsilateral rACC	12	36	20	3.84	82	−0.14 ± 0.06	0.34 ± 0.10	0.02
Ipsilateral dACC	6	15	37	3.71	271	−0.26 ± 0.13	0.59 ± 0.20	0.008
Ipsilateral MCC	4	−16	40	3.62	50	−0.36 ± 0.09	0.32 ± 0.20	0.01
Ipsilateral SMA	4	8	45	4.31	180	−0.47 ± 0.17	0.34 ± 0.19	0.03
Contralateral S1	−38	−34	46	4.10	367	−0.45 ± 0.17	0.39 ± 0.14	0.28

The level of significance between conditions and groups is provided in the right most column. Co-ordinates are in Montreal Neurological Institute (MNI) space. Cluster sizes are derived from resliced 1 mm isotropic image series. “ipsilateral” = right.

*NAcc* nucleus accumbens, *rACC* rostral anterior cingulate cortex, *dACC* dorsal anterior cingulate cortex, *MCC* mid cingulate cortex, *SMA* supplementary motor area, *S1* primary somatosensory cortex.

Similarly to the regions identified in the *stimulus independent network*, significant clusters in the *dependent network* were saved a VOI masks and measures of pain-related connectivity from the placebo nonresponder group as well as signal intensity change from both groups were extracted. Apart from the S1, non-responders displayed no significant change in lPAG-connectivity in any region within this stimulus-dependent network (Supplementary Table 4), nor did any region demonstrate significant differences in functional activation between control- and placebo-site stimulation in either the placebo responder or nonresponder group (Supplementary Table 5).

These findings support the presence of a distinct network of cortical sites which contact brainstem pain-modulatory pathways during periods of pain in the context of placebo. Unlike regions revealed through functional connectivity, the *stimulus dependent network* comprises frontotemporal structures previously identified as activating during the sensory experience of pain and its cognitive modulation^24–26^. However, consistent with the regions revealed through functional connectivity, our findings again indicate that it is primarily via lPAG connectivity, and not via changes in pain-related activation that these regions influence significant placebo analgesia.

### Hypothalamic projections establish and cingulate projections drive brainstem modulatory output to produce placebo analgesia

**Fig. 3:**
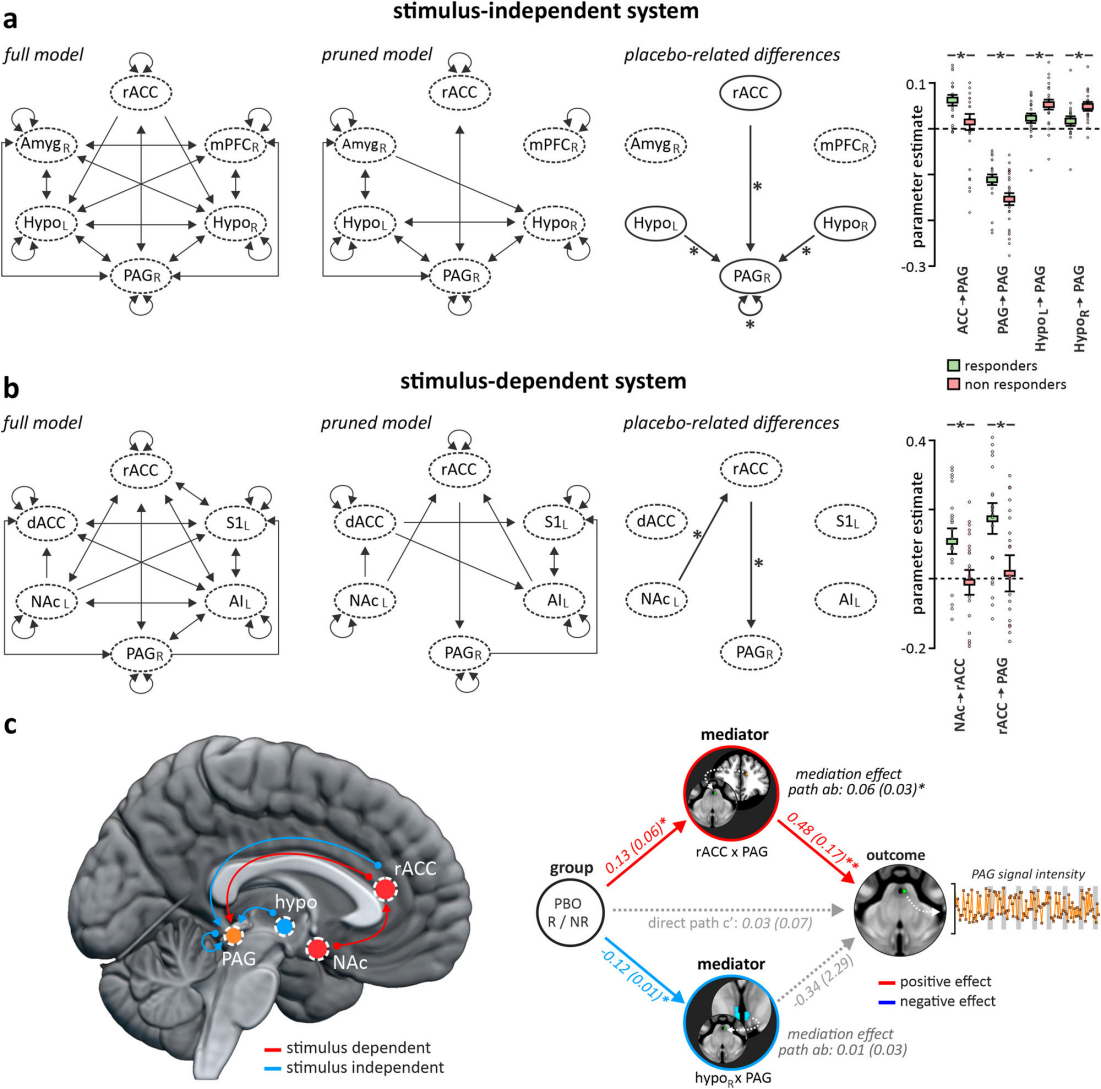
Defining a stimulus-dependent and -independent network of brainstem connectivity. Dynamic Causal Modeling (DCM) was conducted by entering the timeseries of each significant cluster revealed by stimulus dependent (PPI) and -independent (FC) analyses during the stimulation of the placebo lidocaine-site into two, separate, full model designs (*n* = 47). Each anatomically possible connection was turned on to create a full model, and the timing of stimuli was added to the PPI DCM to account for the stimulus dependency of these connections. For both models, DCM was conducted as a bilinear model, one state per region, centered inputs, with stochastic effects off. After specifying and estimating these models, a nested search was conducted revealing connections between clusters whose timeseries significantly added to optimal model evidence. The pruned models displayed in the upper and lower central panels were threshold at *p* > 0.99. Each first-level model was then inspected for each participant, and individual connection parameter estimates were extracted. Two-sample *t*-tests were conducted comparing mean parameter estimates between responders and non-responders to identify cluster connections that significantly differed between placebo responders and non-responders. **a** Stimulus-independent system: Significant differences were identified from the rostral anterior cingulate cortex (rACC), left and right posterior hypothalamus (hypo) to the midbrain periaqueductal gray matter (PAG), as well as in the PAG-PAG self-connection. **b** Stimulus-dependent system: Significant differences were identified from the nucleus accumbens (NAc) to the rACC, and from the rACC to the PAG. **c** View of significant connections in both the stimulus-dependent (red circles and lines), and stimulus-independent (blue circles and lines) networks displaying differences in PAG connectivity between placebo responders and non-responders. Mediation testing revealed that the stimulus-dependent connectivity between the rACC-PAG completely mediated the relationship between placebo responsivity (placebo responder or non-responder group assignment), and PAG signal intensity change. Stimulus independent connectivity between the right hypothalamus-PAG related directly to group assignment, however, did not act as a mediator in the signal change of the PAG. Box plots depict the mean values with individual participant values plotted for each group and connection with error bars representing ±SEM surrounding this mean.

To determine whether regions within the stimulus-independent and stimulus-dependent networks were working collectively, and to determine the direction of information flow, i.e. whether regions within each network were modulating the lPAG or vice versa, we performed a DCM analysis. Each anatomically possible connection, as well as inhibitory self-connections between all regions within each of the two networks were entered as a “full model” (Fig. 3a, b). The timing of noxious stimuli was included in the stimulus-dependent DCM analysis. Model estimation was performed at 256 maximum iterations, after which a nested search identified the combination of anatomical connections which optimized model free energy (i.e. which time-series data best predicted other VOI time-series data in either a forward, or reverse direction). Individual participant parameter estimates were then extracted from each connection which survived the nested search and were inspected for differences between placebo responder and non-responder groups.

Within the *stimulus-independent network*, placebo responses were driven by descending inputs from the left and right PH and the rACC onto the lPAG. These findings are consistent with the idea that reduced drive from the hypothalamus to the lPAG is required for a placebo analgesia to occur (Fig. 3a, Table 3). In addition, a reduced lPAG inhibitory self-connection suggests that in responders, the lPAG is under less inhibitory regulation and thus more capable of being modulated by extrinsic connections^27,28^. Within the *stimulus-dependent network*, differences also occurred in the descending rACC-lPAG connection as well as the NAc-rACC connection (Fig. 3b, Table 3). These data reveal that the rACC regulates the lPAG in both a stimulus-dependent and stimulus-independent manner. Furthermore, these analyses show that within the stimulus-dependent and stimulus-independent networks, the NAc, rACC and PH are the main sites that determine whether an individual will express a placebo analgesic response.

**Table 3:** Significant modulatory parameter estimate means and standard error as determined by nested search dynamic causal modeling (DCM).

Connection	Mean (±SEM) Responder	Mean (±SEM) Nonresponder	Cohen’s D (effect size) 95% CI
Stimulus dependent system			
NAcc → rACC	0.11 ± 0.03	−0.01 ± 0.04	0.76 [0.16–1.37]
rACC → PAG	0.18 ± 0.04	0.02 ± 0.05	0.81 [0.20–1.42]
Stimulus independent system			
Right Hypo → PAG	0.02 ± 0.01	0.05 ± 0.01	0.92 [0.31–1.54]
Left Hypo → PAG	0.02 ± 0.01	0.05 ± 0.01	0.73 [0.12–1.33]
rACC → PAG	0.06 ± 0.01	0.01 ± 0.02	0.68 [0.09–1.29]
PAG → PAG	−0.10 ± 0.01	−0.16 ± 0.01	0.76 [0.16––1.37]

Effect sizes were calculated by Cohen’s D.

To explore the effects of these stimulus-dependent and stimulus-independent network sites on placebo-evoked lPAG signal intensity changes, a dual-path mediation analysis was performed. The rACC-lPAG stimulus-dependent connectivity values and right PH-lPAG stimulus-independent connectivity values were entered as potential mediators of placebo responses and lPAG signal changes. We found that rACC-lPAG stimulus-dependent connectivity completely mediated this relationship, whereas the PH-lPAG connectivity directly related to placebo responsivity (group assignment), but did not drive the changes in lPAG signal intensity (Fig. 3c). These data support our hypothesis that (i) the stimulus-independent network, particularly the PH, sets the sensitivity of the lPAG, whereas (ii) the stimulus-dependent network, particularly top-down communication between the rACC-lPAG, are responsible for driving the output of descending analgesic pathways.

## Discussion

Our results show that placebo analgesia responsivity is regulated by two brain networks, one which sets the sensitivity of the lPAG, and another which drives descending inputs onto the lPAG during noxious stimuli. We propose a stimulus-independent network comprised of the rACC and PH that sets the gain of the lPAG and ultimately whether an individual expresses placebo analgesia. This pathway has previously been described in experimental animals, with prelimbic, hypothalamic and amygdala projections to the lPAG critical for coordinating autonomic and homeostatic processes^23^. An integral part of the active emotional coping behaviors mediated by the lPAG is an analgesia thought to aid an individual’s ability to cope immediately with the source of pain^24^. While analgesia forms a critical part of this primitive behavioral response, it appears that higher brain regions recruit the lPAG pain modulatory circuitry in more abstract situations such as during placebo analgesia. Our results demonstrate that the descending modulatory pathway is at least partially preserved in humans, and that reduced PH-lPAG connectivity likely represents a weakening of PH regulatory grip over the lPAG and disruption to its excitatory-inhibitory balance. This then enables top-down noxious-stimulus evoked modulation of the PAG by regions within the stimulus-dependent network. Importantly, all individuals expect a pain intensity reduction during the placebo scan, however in only those that subsequently mount an analgesic response do changes in rACC-lPAG and PH-lPAG connectivity occur. This suggests that some individuals are set to respond, and others are not, despite having similar expectations. Whether the ability of the PH and rACC to modulate the lPAG is “hard-wired” in an individual, or is shaped by prior experience, influenced by genetic factors, or varies from day to day or between various conditioning effects or environmental associations remains to be determined.

In addition, we reveal a noxious stimulus-dependent network that underpins both lPAG signal intensity changes and placebo responses. Whilst this network consisted of multiple higher order processing regions such as the dACC, MCC, and insula, the rACC appears to be critical in mediating both placebo responsivity and lPAG signal intensity changes. Indeed, prior investigations have demonstrated that heightened rACC-PAG coupling underlies placebo responses in acute settings^5,25^. Additionally, reductions in analgesic phenomena in response to naloxone (opioid antagonist) administration have been consistently tied with reductions in rACC-PAG coupling^7,26^. The NAc was also identified as part of our stimulus-dependent network. Forming part of the ventral striatum and acting as a cortical dopaminergic hub, the NAc contacts the prefrontal cortex to drive reward-anticipation, decision-making, and error-predictions^27^. Correcting perception-anticipation differentials is a critical component in mounting placebo analgesic responses, and one which has been associated with activation and neurotransmission within the NAc and its cortical efferents^27,28^. Our data shows that during noxious stimulation, phasic coupling between the NAc and rACC are critical for lPAG ability to drive analgesic responses and match anticipated pain.

Although we utilized PPI, FC, and directed connectivity analyses to unveil the most integral cortical networks that regulate and drive PAG output during placebo analgesia – these networks were identified by considering the phenomena as dichotomous. That is, we delineated and investigated a responder and non-responder group. Performing these analyses allowed us first to identify which cortical connections with the PAG were significantly altered in those demonstrating a placebo response as determined through the 2SD band method, and then assess these connections in non-responders for statistical differences. There exists conflicting literature over the method of determining placebo responses (for example by using arbitrary VAS changes or permutation testing), as well as if placebo analgesia should be considered a continuous variable^29–31^. Whilst these approaches are well- documented, the former is limited by not considering an individual’s perceived pain intensity baseline variability, and the latter would only identify clusters where PAG connectivity shows a linear relationship with graded changes in perceived pain – including in those where pain either did not change between the control vaseline- and placebo lidocaine-sites or indeed in those who’s pain increased when exposed to placebo. As such, in our analysis we can be confident that we have described a functional architecture underpinning significant placebo analgesia, and that these same connections are unchanged in non-responders.

Additionally, as we conducted our DCM and mediation analyses using clusters that were first revealed using the lPAG timeseries as a seed, our results are constrained to solely regions which likely receive information from or project directly to the lPAG. Whilst this does not allow assessment of regions which comprise alternate projection pathways which may be involved in the response, encoding more nuanced aspects of placebo analgesia such as cognitive evaluation or complex emotional processing, the results presented do offer valuable insight into the functional projections regulating and driving brainstem output in humans to produce an antinociceptive state.

It is of note that despite receiving an identical response conditioning protocol, over half our sample did not demonstrate a significant placebo response. Whilst the focus of this investigation was to identify the functional networks of placebo responders, it would also be of interest to better understand the driving factors influencing why certain individuals fail to generate analgesic responses to placebo. Two leading theories – the Bayesian brain hypothesis^32^ and an individual’s underlying biological substrates^18^ both center on error-prediction signaling and dopaminergic neurotransmission, which encompass roles of the NAc. Since we identified this region as feeding into the rACC-PAG pathway in a stimulus-dependent manner, it may be that this cortical site is a key delineating factor between an individual forming accurate stimulus-response relationships and generating placebo responses via response conditioning. Future studies could compliment this work by assessing the role of the NAc in the conditioning phases of placebo analgesia, and indeed whether NAc activation or neurotransmission during these phases could be a potential biomarker of placebo responsiveness.

In conclusion, we provide evidence for two brain networks responsible for altering descending brainstem pathways during placebo analgesia. Whilst this investigation utilized a specific protocol consisting of short lasting thermal stimuli and response conditioning to induce analgesic effects, regions in the network we describe have previously been tied with analgesia elicited from longer lasting stimuli or chronic conditions^7,33^. Recently, brainstem projections from the hypothalamus have been linked to pain anticipation in Fibromyalgia patients^34^, and the cingulate cortex – specifically it’s anterior division - has been proposed as a neurosurgical target to treat intractable pain due to its role in emotional and attentional processing during painful events^35^. Additionally, the same regions we identify from placebo analgesia generated by response conditioning appear to be involved in alternative placebo substances such as social observation which includes the amygdala and PAG^36^, and pharmacological conditioning which include the rACC and PAG^37^. These data are consistent with frontotemporal and limbic structures playing a generalized role in recruiting brainstem pain-modulatory circuits to drive analgesia, emphasizing the role of “mind set” and emotion in influencing our responses to pain. Indeed, it remains to be seen whether these specific connections between cortical, subcortical, and brainstem sites are compromised in individuals with chronic pain or underpin alternative endogenous pain modulatory phenomena.

## Methods

### Ethics

All experimental procedures were approved by the University of Sydney Human Research Ethics Committee and were consistent with the Declaration of Helsinki. Written informed consent was obtained from participants at the commencement of the study. Participants were also provided with an emergency buzzer while inside the scanner so that they could stop the experiment at any time. At the conclusion of testing, participants were informed both verbally and through a written statement of the necessary deception and true methodology of the experiment and were invited to seek clarification of what they had just experienced.

### Participants

Forty-seven healthy control participants were recruited for the study (25 male, 22 female; mean age, 24.0 ± 0.5 years [±SEM]; range 19–37 years). In order to evaluate the necessary number of participants required for this study, an *apriori* power analysis was performed using results from a previous imaging study investigating cortico-brainstem connectivity during placebo analgesia^7^. This revealed a total sample size of 40 would be necessary to detect similar effect sizes with 95% power (d = 0.31, α = 0.05, power = 0.95). Before beginning the study, participants completed a data sheet recording current medication(s), and any alcohol or caffeine ingested in the 24 h prior to testing.

### Experimental design

The study included three sessions occurring on two successive days: a conditioning session on day 1, and a reinforcement and MRI scanning session on day 2 (Fig. 1a). Throughout the study, noxious stimuli were administered to the volar surfaces of participants’ left and right forearms using a 3 × 3 cm MR-compatible Peltier element thermode, which delivered a heat stimulus at a pre-programmed temperature via a Thermal Sensory Analyzer (TSA-II) (Medoc LTD Advanced Medical Systems, Rimat Yishai, Israel). Each stimulus lasted 15 s, including a ramp-up period (four degrees per second), a plateau period at a noxious temperature and a ramp-down period (four degrees per second). Each stimulus was separated by a 15 s inter-stimulus-interval (ISI) at a non-painful baseline temperature of 32 °C. Throughout conditioning, participants rated their pain on-line using a horizontal 10 cm visual analog scale (VAS) ranging between 0 and 100, where 0 was described as “no pain” and 100 as “the worst pain imaginable”. During scanning, participants used an MR-compatible button box to continuously report their pain perception. The VAS scale was shown on a reflected digital screen at the end of the magnet bore, and participants controlled the position of a slider to report their pain continuously by holding the left (moved slider towards zero) or right (moved slider towards ten) buttons with their left middle and index finger.

### Conditioning

Session 1 was conducted outside the MRI and consisted of two rounds of a conditioning protocol. Participants were first informed both verbally and via a written statement that the study was designed to investigate the modulatory effects of a topical anesthetic containing lidocaine, which had been shown to provide pain relief in some individuals. A second control cream was stated to be purely vaseline and was stated as being necessary to evaluate typical pain responses. In reality, both creams contained vaseline and only differed in color and their described properties. We calculated individual low and moderate pain responses by applying a series of randomized stimuli to the left forearm ranging from 44 to 48.5 degrees, asking participants to rate their perceived pain during each stimulus. Participants were informed that we were only recording a temperature which elicited a moderate subjective pain response (40–50 VAS rating), and that this temperature would be used throughout the remainder of the experiment. However, using the ratings provided during this process, we recorded two different temperatures: one which was rated between 20 and 30 on the VAS (low temperature); and one which was rated between 40 and 50 (moderate temperature). These two temperatures were then deceptively applied to the “lidocaine” and vaseline cream sites throughout the conditioning and reinforcement experimental phases.

Creams were then applied to two adjacent 3 × 3 cm squares on the volar surface of the participants’ right forearm. To enhance the believability that the “lidocaine” cream contained an active analgesic, a false label was attached to the cream bottle and green food coloring was added. The positions of the “lidocaine” and vaseline creams were counterbalanced between proximal and distal sites on the volar right forearm between participants to reduce potential confounders of local sensitivity, however we ensured both creams always occupied the C6 dermatome. Ten minutes following cream application, we conducted two rounds of conditioning. Participants believed they would receive eight identical moderate thermal stimuli and were instructed to report their perceived pain intensity using the VAS. Participants were also asked prior to each set of stimuli for an average expectation of the pain they would experience, which acted both to measure belief that lidocaine was working to modulate their subjective pain, and to reinforce the pain relieving quality of the cream. During the two conditioning rounds we deceptively applied a moderate temperature to the control vaseline-site, and a low temperature to the placebo lidocaine-site.

### Reinforcement and test

At approximately the same time on the following day, sessions 2 and 3 were conducted with participants inside the MRI scanner and consisted of a reinforcement protocol (session 2) and a test protocol (session 3). The creams were applied to the volar surface of both left and right forearms, in the same order and locations as session 1, and participants were reminded of the “lidocaine’s” pain-relieving qualities. To ensure that the protocol for conditioning was consistent between subsequent days, and the change in immediate environment (inside the MRI), reinforcement was conducted by applying four noxious stimuli at the same low and moderate temperatures used throughout session 1 to participants’ left volar forearm. This was performed on the opposite forearm to prevent sensitization of the testing area (the right volar forearm).

Following reinforcement, we waited 15 min for residual pain and sensitivity to dissipate from the left arm before beginning the test protocol. During this 15-min period, structural brain scans were collected. Dissimilar to conditioning and reinforcement, during the test phase we applied *identical moderate temperature stimuli* to both the control vaseline- and placebo lidocaine-sites (Fig. 1a). We asked each participant for an average expectation of pain intensity directly prior to each stimulation series and instructed them to report the pain intensity continuously throughout the duration of the scan using the button box and the projected digital VAS. VAS responses were recorded every 0.5 s, and values during each pain period were averaged providing a pain intensity for each noxious stimulus period. Each participant received two consecutive series of eight stimuli, with a separate functional series collected during each series of stimuli. Each fMRI series began with a 90-s baseline period prior to the eight stimuli presentations. The control vaseline-site was always stimulated during the first series, and the placebo lidocaine-site was stimulated during the second series, so that we generated a “pre” and “post” condition, or, functional brain images encoding typical and placebo pain responses, respectively.

### MRI data acquisition and preprocessing

Brain images were acquired using a whole body Siemens MAGNETOM 7 Tesla (7T) MRI system (Siemens Healthcare, Erlangen, Germany) with a combined single-channel transmit and 32-channel receive head coil (Nova Medical, Wilmington MA, USA). Participants were positioned supine with their head in the coil and sponges supporting the head laterally to minimize movement. A T1-weighted anatomical image set covering the whole brain was collected (repetition time = 5000 ms, echo time = 3.1 ms, raw voxel size = 0.73 × 0.73 × 0.73 mm, 224 sagittal slices, scan time = 7 min). The two fMRI acquisitions each consisted of a series of 134 gradient echo echo-planar measurements using blood oxygen level dependent (BOLD) contrast covering the entire brain. Images were acquired in an interleaved collection pattern with a multi-band factor of four and an acceleration factor of three (repetition time = 2500 ms, echo time = 26 ms; raw voxel size = 1.0 × 1.0 × 1.2 mm, 124 axial slices, scan time = 5:35 min).

Image preprocessing and statistical analyses were performed using SPM12^38^ and custom software. The first five volumes of each scan were removed from the model due to excessive signal saturation from the scanner. The remaining 129 functional images were slice-time and motion corrected and the resulting 6 directional movement parameters were inspected to ensure that all fMRI scans had no greater than 1 mm of linear movement or 0.5 degrees of rotation movement in any direction. In no single participant in either the placebo lidocaine- or control vaseline-site scans did motion parameters exceed our elected threshold. Images were then linearly detrended to remove global signal changes, physiological noise relating to cardiac (frequency band of 60–120 beats per minute +1 harmonic) and respiratory (frequency band of 8–25 breaths per minute +1 harmonic) frequency was removed using the DRIFTER toolbox^39^, and the 6-parameter movement related signal changes were modeled and removed using a linear modeling of realignment parameters (LMRP) procedure. Each individual’s fMRI image sets were then coregistered to their own T1-weighted anatomical, the T1 was then spatially normalized to the DARTEL template in Montreal Neurological Institute (MNI) space and the parameters applied to the fMRI image sets. The normalized fMRI images were then spatially smoothed using a 6 mm full-width at half maximum Gaussian filter.

### Dichotomizing placebo responder and non-responder groups

Participants were grouped as either a responder or non-responder to placebo analgesia based on the two-standard deviation (SD) method described previously^19^. Briefly, for the 8 noxious stimuli delivered during the test phase to the control vaseline-site, the SD of the 8 pain intensity ratings was calculated. During the stimulation of the placebo lidocaine-site, the average pain intensity rating was calculated, and if this average rating was 2 SD lower than the control vaseline-site, the participant was considered a responder. If not, they were considered a non-responder (Supplementary Table 6). Significant differences between groups with respect to expected changes in pain intensities immediately prior to testing were determined using paired *t*-tests (two-tailed, *p* < 0.05). Since participants were grouped into either responder or non-responder categories based on their perceived pain intensities during the fMRI scans (session 3), we did not assess significant differences between groups for the perceived pain intensity changes. A single factor ANOVA (*p* < 0.05) was used to determine if there were differences in the temperature applied or pain intensity ratings reported between responder and non-responder groups during the control stimulated series to ensure any reported placebo effects did not relate to baseline thermal sensitivity.

### PAG region-of-interest generation

Previously, we identified a region of the caudal lateral PAG (lPAG) ipsilateral to the side of stimulation as primarily responsible for placebo analgesia^8^. We began by running a two-sample difference map between control vaseline and placebo lidocaine-site scans between placebo responder and non-responder groups and confirmed that the greatest change in placebo-related activity occurred at the same lPAG location as that reported earlier. We generated a 1 mm radius spherical volume of interest mask (VOI) at this lPAG site and used this VOI throughout subsequent connectivity analyses to assess changes in stimulus-dependent and -independent cortical coupling with the PAG during significant placebo responses (Fig. 1d).

### fMRI statistical analysis

To determine significant changes in signal intensity during each noxious thermal period, a repeating boxcar model convolved with a canonical hemodynamic response function was applied to each of the fMRI series. Within this model, scanning volumes overlying stimulus plateau periods were assigned a value of 1, and inter-stimulus-intervals and the initial 90 s baseline period were assigned a value of 0. The contrast images generated for each functional image series were then used in group analyses. We conducted four separate analyses to determine the cortical constituents of placebo analgesia and brainstem engagement.

*Analysis 1*: cortico-PAG stimulus independent connectivity changes in responders and non-responders were assessed by conducting a functional connectivity (FC) analysis. This analysis generates contrast images with includes the timeseries of the PAG seed as a regressor, independent to the timing of noxious stimuli applied (Fig. 2a). As such, this analysis reveals cortical regions contacting the PAG during the entire scan period including the baseline anticipation, pain, ramp, and inter-stimulus-interval periods. Using these contrast images, a random-effects paired, voxel-by-voxel analysis was conducted in placebo responders comparing the control vaseline- and placebo lidocaine-site series. From resulting clusters, eigenvariates representing stimulus-independent connectivity with the PAG in each series were extracted from both placebo responder and non-responder contrast images and significance was determined in both groups using paired *t*-tests (Fig. 2b, c). To determine whether these changes in connectivity were significantly difference between placebo responder and nonresponder groups, post minus pre connectivity difference values were generated for each group, and two sample *t*-tests performed (*p* < 0.05) (Table 1).

*Analysis 2:* cortico-PAG stimulus dependent connectivity changes in responders and non-responders were assessed by conducting a psychophysiological interaction (PPI) analysis. This analysis involves extracting the timeseries of the PAG from each subject’s control vaseline- and placebo lidocaine-site scans and convolving it with the repeating boxcar model which isolates scan periods in which a noxious stimulus was applied. This generates a new stimulus*PAG timeseries regressor which is then applied to functional series to create new contrast images of stimulus-dependent PAG connectivity (Fig. 2c). Using these contrast images, a random-effects paired, voxel-by-voxel analysis was conducted in placebo responders comparing the control vaseline- and placebo lidocaine-site scans. From resulting clusters, eigenvariates representing stimulus-dependent connectivity with the PAG in each series were extracted from both placebo responder and non-responder contrast images and significance was determined in both groups using paired *t*-tests (Fig. 2e, f). To determine whether these changes in connectivity were significantly difference between placebo responder and nonresponder groups, post minus pre connectivity difference values were generated for each group, and two sample *t*-tests performed (*p* < 0.05) (Table 2).

*Analysis 3:* network properties and directed connectivity in PPI and FC clusters were compared by conducting two separate Dynamic Causal Modeling (DCM) analyses. DCM is a technique whereby cluster timeseries are compared to consider is a region’s activity over time can be used to predict the activity in a second, connected region. After entering an appropriate “full model” which includes all anatomically possible connections as well as inhibitory self-connections between each entered cluster, a nested search step can be performed which sequentially tests combinations of connections to produce the most likely “reduced model”, or, the combination of connections which maximises free energy (Fig. 3a)^22^. We conducted our two DCM’s using the following parameters: slice timing = 1.25 s (modeled to the center slice of acquisition), echo time = 0.026 s, bilinear modulatory effects, one state per region, stochastic effects off, centered inputs on, and a timeseries fit. The timings of noxious stimuli were modeled specifically in the PPI DCM and added as potential contributors to all extrinsic and intrinsic connections of the full model due to the inherent stimulus-dependency of these clusters. After identifying the optimal reduced model through nested search, individual participant parameter estimates for each resulting between-cluster and self-connection were extracted and effect sizes and 95% confidence intervals calculated by Cohen’s d to identify directed connections with medium to large effects between placebo responders and non-responders (Cohens *d* > 0.5).

*Analysis 4:* potential cortical mediators of placebo responsiveness and PAG activation were investigated by entering the most pronounced connection elucidated from the two DCM analyses into a multiple mediation analysis performed using the Canlab Mediation Toolbox in Matlab R2022b^40^. Mediation analyses are routinely used to investigate if the relationship between two variables is direct, or reliant on a third, contingent variable. In our investigation, we entered “placebo responsiveness” as the input variable (X), and PAG signal intensity change as the output variable (Y). Connectivity between the rACC-PAG in the PPI analysis, and right hypothalamus-PAG in the FC analysis were entered as potential mediators (M1 and M2, respectively).

Analyses 1 and 2 were initially visualized at a threshold of *p* < 0.001 uncorrected with a cluster extent threshold of 20 contiguous voxels. We then applied small volume correction (*p* < 0.05) to reduce the likelihood of type II errors. The VOI used to perform these small volume corrections were derived from parcels in the extended human connectome project atlas (HCPex) which includes subcortical areas such as the amygdala and hypothalamus^41^. The locations of significant clusters in MNI space were tabulated and beta-values extracted to determine the directions of signal and PAG-connectivity change. For display purposes, significant clusters were overlaid onto a mean T1 weighted anatomical of all 47 participants. For Analysis 3, posterior probabilities of the reduced model after nested search were thresholded at *p* > 0.99, and effect sizes of parameter estimate differences between responders and non-responders were discerned using Cohen’s d tests. Analysis 4 was performed at a false discovery rate correction of *p* < 0.05, bootstrapped to 10,000 samples.

### Reporting summary

Further information on research design is available in the Nature Portfolio Reporting Summary linked to this article.

## Supplementary information

Peer Review File

Supplementary Information

Description of Additional Supplementary Files

Supplementary Data 1

Reporting Summary

## Data Availability

All de-identified single participant functional data, as well as activation and connectivity contrast maps are available from the corresponding author upon reasonable request. Source data used to generate the results found in Figs. 1, 2, and 3 is available with the manuscript as Supplementary Data 1.

## Code availability

The analysis methods and software used in this article are all either open source (Canlab mediation toolbox - https://github.com/canlab/MediationToolbox) or enabled in SPM12’s standard installation. For this manuscript, MATLAB version R2022b was used. No new methods or algorithms have been generated.

## References

[1] Kirsch I (2014). Expectancy and conditioning in placebo analgesia: separate or connected processes?. Psychol. Conscious.

[2] Medoff ZM, Colloca L (2015). Placebo analgesia: understanding the mechanisms. Pain. Manag..

[3] Tracey I, Mantyh PW (2007). The cerebral signature for pain perception and its modulation. Neuron.

[4] Petrovic P, Kalso E, Petersson KM, Ingvar M (2002). Placebo and opioid analgesia- imaging a shared neuronal network. Science.

[5] Schafer SM, Geuter S, Wager TD (2018). Mechanisms of placebo analgesia: a dual-process model informed by insights from cross-species comparisons. Prog. Neurobiol..

[6] Wager TD (2004). Placebo-induced changes in FMRI in the anticipation and experience of pain. Science.

[7] Eippert F (2009). Activation of the opioidergic descending pain control system underlies placebo analgesia. Neuron.

[8] Crawford LS (2021). Brainstem mechanisms of pain modulation: a within-subjects 7T fMRI study of placebo analgesic and nocebo hyperalgesic responses. J. Neurosci..

[9] Mokhtar, M. & Singh, P. *Neuroanatomy, periaqueductal gray* (StatPearls, 2020). 32119278

[10] Ellison GD, Flynn JP (1968). Organized aggressive behavior in cats after surgical isolation of the hypothalamus. Arch. Ital. Biol..

[11] Eclancher F, Schmitt P (1972). [Effect of early lesions of the amygdala and median hypothalamus on the development of interspecific aggression behavior in rats]. J. Physiol..

[12] Huang YH, Flynn JP (1975). Unit activities in the hypothalamus and midbrain during stimulation of hypothalamic attack sites. Brain Res..

[13] Wang W (2021). Coordination of escape and spatial navigation circuits orchestrates versatile flight from threats. Neuron.

[14] Stroman PW, Ioachim G, Powers JM, Staud R, Pukall C (2018). Pain processing in the human brainstem and spinal cord before, during, and after the application of noxious heat stimuli. Pain.

[15] Craig AD (2003). Interoception: the sense of the physiological condition of the body. Curr. Opin. Neurobiol..

[16] Eippert F, Finsterbusch J, Bingel U, Büchel C (2009). Direct evidence for spinal cord involvement in placebo analgesia. Science.

[17] Wager TD, Scott DJ, Zubieta JK (2007). Placebo effects on human mu-opioid activity during pain. Proc. Natl Acad. Sci. USA.

[18] Scott DJ (2008). Placebo and nocebo effects are defined by opposite opioid and dopaminergic responses. Arch. Gen. Psychiatry.

[19] Nourbakhsh MR, Ottenbacher KJ (1994). The statistical analysis of single-subject data: a comparative examination. Phys. Ther..

[20] Necka, E. A. et al. Applications of dynamic functional connectivity to pain and its modulation. *PAIN Rep.* **4**, e752 (2019). 10.1097/PR9.0000000000000752 PMC6728009 31579848

[21] Snyder, A. D., Ma, L., Steinberg, J. L., Woisard, K. & Moeller, F. G. Dynamic causal modeling self-connectivity findings in the functional magnetic resonance imaging neuropsychiatric literature. *Front. Neurosci.* **15**, 10.3389/fnins.2021.636273 (2021). 10.3389/fnins.2021.636273 PMC8385130 34456665

[22] Friston KJ, Harrison L, Penny W (2003). Dynamic causal modelling. NeuroImage.

[23] Ongür D, An X, Price JL (1998). Prefrontal cortical projections to the hypothalamus in macaque monkeys. J. Comp. Neurol..

[24] Bandler R, Shipley MT (1994). Columnar organization in the midbrain periaqueductal gray: modules for emotional expression. Trends Neurosci..

[25] Bingel U, Lorenz J, Schoell E, Weiller C, Buchel C (2006). Mechanisms of placebo analgesia: rACC recruitment of a subcortical antinociceptive network. Pain.

[26] Oliva V (2021). Parallel cortical-brainstem pathways to attentional analgesia. NeuroImage.

[27] Benedetti F, Amanzio M (2013). Mechanisms of the placebo response. Pulm. Pharmacol. Therapeutics.

[28] Scott DJ (2007). Individual differences in reward responding explain placebo-induced expectations and effects. Neuron.

[29] Jensen KB (2015). A neural mechanism for nonconscious activation of conditioned placebo and nocebo responses. Cereb. Cortex.

[30] Kaptchuk TJ (2008). Do “placebo responders” exist?. Contemp. Clin. Trials.

[31] Olsen MF (2018). Minimum clinically important differences in chronic pain vary considerably by baseline pain and methodological factors: systematic review of empirical studies. J. Clin. Epidemiol..

[32] Pagnini, F. et al. Placebo and nocebo effects as Bayesian-brain phenomena: the overlooked role of likelihood and attention. *Perspect. Psychol. Sci.*, 17456916221141383, 10.1177/17456916221141383 (2023). 10.1177/17456916221141383 36656800

[33] Schmid J (2015). Placebo analgesia in patients with functional and organic abdominal pain: a fMRI study in IBS, UC and healthy volunteers. Gut.

[34] Ioachim G (2022). Altered pain in the brainstem and spinal cord of fibromyalgia patients during the anticipation and experience of experimental pain. Front. Neurol..

[35] Peyron, R., Quesada, C. & Fauchon, C. In *Handbook of Clinical Neurology* Vol. 166 (ed Brent, A. V.) 317–326 (Elsevier, 2019). 10.1016/B978-0-444-64196-0.00017-0 31731919

[36] Schenk, L. A. & Colloca, L. The neural processes of acquiring placebo effects through observation. *Neuroimage*, 116510, 10.1016/j.neuroimage.2019.116510 (2019). 10.1016/j.neuroimage.2019.116510 PMC7107761 31899287

[37] Vachon-Presseau E (2018). Brain and psychological determinants of placebo pill response in chronic pain patients. Nat. Commun..

[38] Penny, W. D., Friston, K. J., Ashburner, J. T., Kiebel, S. J. & Nichols, T. E. *Statistical parametric mapping: the analysis of functional brain images* (Elsevier, 2011).

[39] Särkkä S (2012). Dynamic retrospective filtering of physiological noise in BOLD fMRI: DRIFTER. NeuroImage.

[40] Wager TD (2009). Brain mediators of cardiovascular responses to social threat, part II: prefrontal-subcortical pathways and relationship with anxiety. Neuroimage.

[41] Huang CC, Rolls ET, Feng J, Lin CP (2022). An extended Human Connectome Project multimodal parcellation atlas of the human cortex and subcortical areas. Brain Struct. Funct..

