# Supplementary material for: Stimulus-independent and stimulus-dependent neural networks underpin placebo analgesia responsiveness in humans
Source: Commun Biol. 2023 May 27;6:569. doi: 10.1038/s42003-023-04951-7 (PMC10224990; doi:10.1038/s42003-023-04951-7)
Supplement: Supplementary file 2 — Supplementary Information [file 42003_2023_4951_MOESM2_ESM.pdf]

Supplementary Information:

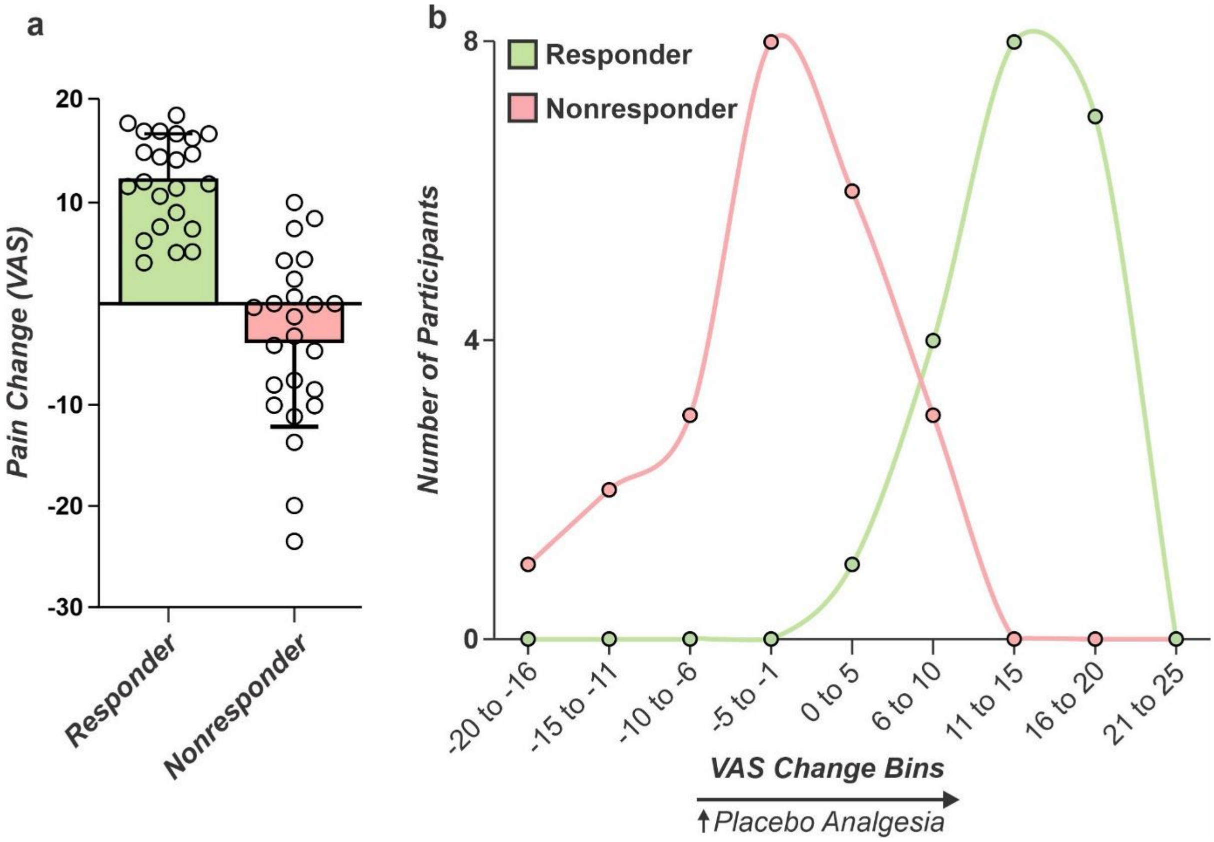

Supplementary Figure 1. Individual participant pain change data and placebo analgesia distribution. a) Placebo responsiveness. Based on the two standard deviation band method, placebo responders and non-responders were delineated and changes in visual analogue scale (VAS) values determined. More positive values indicate a greater placebo response when both control vaseline- and placebo lidocaine-sites received identical intensity stimuli during the test phase. **b) Distinct group-level difference in placebo analgesia distribution.** Evenly spaced bins covering the extent of VAS changes in all participants were generated and a count of participants satisfying the criteria of these bins in both placebo responder (green curve, n=23) and nonresponder (pink curve, n=24) groups were determined. More positive values indicate a greater placebo analgesia response.

Supplementary Table 1. Temperatures applied throughout conditioning and test phases in placebo responder and nonresponder groups.

	Responder	Nonresponder	P-value
Moderate Temperature (degrees±SEM)	46.8±0.17	46.7±0.20	0.94
Low Temperature (degrees±SEM)	45.8±0.17	45.6±0.22	0.57

Supplementary Table 2. Placebo non responder functional connectivity values in significant clusters of the stimulus independent network.

	PAG Whole scan connectivity change (mean ± SEM)		
	control scan	lidocaine scan	P-value
Functional Connectivity (FC) PBO>Control			
Ipsilateral rACC	0.023±0.010	0.019±0.001	0.84
Contralateral dlPFC	0.014±0.005	0.011±0.006	0.14
PBO>Control			
Ipsilateral mPFC	0.008±0.006	0.010±0.009	0.84
Ipsilateral MeA	0.017±0.007	0.014±0.005	0.68
Ipsilateral Posterior Hypothalamus	0.026±0.010	0.015±0.007	0.30
Contralateral Posterior Hypothalamus	0.024±0.010	0.009±0.006	0.14

Supplementary Table 3. Placebo responder and non-responder signal intensity change values in significant clusters of the stimulus independent network.

	Responder Signal Intensity Change (mean ± SEM)			Non-responder Signal Intensity Change (mean ± SEM)		
	control scan	lidocaine scan	P-value	control scan	lidocaine scan	P-value
Functional Connectivity (FC) PBO>Control						
Ipsilateral rACC	-0.12±0.34	-0.08±0.34	0.86	-0.16±0.18	-0.19±0.19	0.88
Contralateral dlPFC	0.13±0.10	0.19±0.09	0.47	0.10±0.09	0.05±0.09	0.46
PBO>Control						
Ipsilateral mPFC	-0.11±0.09	-0.15±0.09	0.74	-0.09±0.10	-0.09±0.06	0.99
Ipsilateral MeA	0.22±0.12	0.32±0.14	0.51	0.05±0.12	0.16±0.07	0.36
Ipsilateral Posterior Hypothalamus	-0.24±0.22	-0.08±0.28	0.57	-0.08±0.15	-0.08±0.13	0.99
Contralateral Posterior Hypothalamus	0.08±0.21	0.42±0.27	0.22	0.32±0.11	0.14±0.15	0.36

Supplementary Table 4. Placebo non responder psycho-physiological interaction values in significant clusters of the stimulus dependent network.

	PAG Stimulus-dependent connectivity change (mean ± SEM)		
	control scan	lidocaine scan	P-value
Psychophysiological Interaction (PPI) PBO>control			
Contralateral Anterior Insula	-0.31±0.11	-0.17±0.14	0.40
Contralateral NAcc	0.07±0.09	-0.05±0.06	0.35
Ipsilateral rACC	-0.04±0.06	0.07±0.07	0.22
Ipsilateral dACC	-0.29±0.12	-0.03±0.07	0.22
Ipsilateral MCC	-0.25±0.12	-0.04±0.16	0.52
Ipsilateral SMA	-0.24±0.14	0.05±0.17	0.11
Contralateral S1	-0.28±0.13	0.38±0.13	0.004

Supplementary Table 5. Placebo responder and non-responder signal intensity change values in significant clusters of the stimulus dependent network.

	Responder Signal Intensity Change (mean ± SEM)			Non-responder Signal Intensity Change (mean ± SEM)		
	control scan	lidocaine scan	P-value	control scan	lidocaine scan	P-value
Psychophysiological Interaction (PPI) *PBO>control*						
Contralateral Anterior Insula	1.28±0.22	1.29±0.23	0.93	1.07±0.26	1.25±0.22	0.54
Contralateral NAcc	0.05±0.10	0.19±0.10	0.36	-0.01±0.07	-0.02±0.08	0.92
Ipsilateral rACC	0.09±0.15	0.18±0.16	0.34	-0.10±0.11	-0.01±0.12	0.38
Ipsilateral dACC	1.85±0.34	1.73±0.34	0.65	1.47±0.18	1.47±0.20	0.99
Ipsilateral MCC	0.05±0.27	-0.06±0.27	0.64	0.22±0.21	0.19±0.13	0.89
Ipsilateral SMA	2.79±0.39	2.42±0.35	0.29	1.45±0.18	1.45±0.20	0.99
Contralateral S1	0.58±0.20	0.40±0.21	0.43	0.34±0.38	0.16±0.27	0.70

Supplementary Table 6. Demographic data of each participant's age, gender, and group assignment for placebo responders and non-responders.

Responders n=23			Non-responders n=24		
Subject ID	Age	Gender	Subject ID	Age	Gender
2013	20	M	2009	20	M
2015	19	F	2010	20	F
2022	20	F	2011	20	M
2025	22	F	2014	21	M
2028	24	F	2016	19	F
2035	24	F	2021	24	F
2036	21	M	2023	22	M
2037	21	M	2024	21	M
2039	32	F	2026	33	M
2049	23	M	2027	24	M
2056	24	M	2029	27	F
2078	24	F	2030	23	F
2079	25	M	2032	20	F
2082	29	M	2034	23	F
2083	21	F	2040	26	M
2084	25	M	2041	22	M
2085	24	M	2077	23	F
2086	32	F	2080	24	F
2087	25	M	2081	31	M
2088	25	M	2091	23	M
2089	25	F	2095	27	F
2090	24	M	2097	23	F
2094	23	M	2098	23	M
			2099	37	F
Mean±SEM	24.0±0.68	13M:10F		24.0±0.84	12M:12F
