# Supplementary material for: Stimulus-independent and stimulus-dependent neural networks underpin placebo analgesia responsiveness in humans
Source: Commun Biol. 2023 May 27;6:569. doi: 10.1038/s42003-023-04951-7 (PMC10224990; doi:10.1038/s42003-023-04951-7)
Supplement: Supplementary file 3 — Description of Additional Supplementary Files [file 42003_2023_4951_MOESM3_ESM.pdf]

Description of Additional Supplementary Files

File name: Supplementary Data 1

Description: Numerical raw data used to generate results in the paper
