# Supplementary material for: Stimulus-independent and stimulus-dependent neural networks underpin placebo analgesia responsiveness in humans
Source: Commun Biol. 2023 May 27;6:569. doi: 10.1038/s42003-023-04951-7 (PMC10224990; doi:10.1038/s42003-023-04951-7)
Supplement: Supplementary file 5 — Reporting Summary [file 42003_2023_4951_MOESM5_ESM.pdf]

nature portfolio

Corresponding author(s): Luke A Henderson

Last updated by author(s): May 16, 2023

Reporting Summary

Nature Portfolio wishes to improve the reproducibility of the work that we publish. This form provides structure for consistency and transparency in reporting. For further information on Nature Portfolio policies, see our Editorial Policies and the Editorial Policy Checklist.

Statistics

For all statistical analyses, confirm that the following items are present in the figure legend, table legend, main text, or Methods section.

n/a	Confirmed	
☐	☒	The exact sample size (*n*) for each experimental group/condition, given as a discrete number and unit of measurement
☐	☒	A statement on whether measurements were taken from distinct samples or whether the same sample was measured repeatedly
☐	☒	The statistical test(s) used AND whether they are one- or two-sided<br>*Only common tests should be described solely by name; describe more complex techniques in the Methods section.*
☐	☒	A description of all covariates tested
☐	☒	A description of any assumptions or corrections, such as tests of normality and adjustment for multiple comparisons
☐	☒	A full description of the statistical parameters including central tendency (e.g. means) or other basic estimates (e.g. regression coefficient) AND variation (e.g. standard deviation) or associated estimates of uncertainty (e.g. confidence intervals)
☐	☒	For null hypothesis testing, the test statistic (e.g. *F*, *t*, *r*) with confidence intervals, effect sizes, degrees of freedom and *P* value noted<br>*Give P values as exact values whenever suitable.*
☒	☐	For Bayesian analysis, information on the choice of priors and Markov chain Monte Carlo settings
☒	☐	For hierarchical and complex designs, identification of the appropriate level for tests and full reporting of outcomes
☐	☒	Estimates of effect sizes (e.g. Cohen's *d*, Pearson's *r*), indicating how they were calculated

Our web collection on statistics for biologists contains articles on many of the points above.

Software and code

Policy information about availability of computer code

Data collection	Human brain imaging performed on a Siemens MAGNETOM 7 Tesla MRI scanner with a 32-channel receive head coil
Data analysis	Functional brain data preprocessed in Statistical Parametric Mapping version 12 (SPM12). Connectivity (Functional and Psychophysiological interaction) conducted in SPM12. Mediation conducted using Canlab mediation toolbox. Dynamic Causal Modelling conducted using SPM12.

For manuscripts utilizing custom algorithms or software that are central to the research but not yet described in published literature, software must be made available to editors and reviewers. We strongly encourage code deposition in a community repository (e.g. GitHub). See the Nature Portfolio guidelines for submitting code & software for further information.

Data

Policy information about availability of data

All manuscripts must include a data availability statement. This statement should provide the following information, where applicable:

- Accession codes, unique identifiers, or web links for publicly available datasets
- A description of any restrictions on data availability
- For clinical datasets or third party data, please ensure that the statement adheres to our policy

All de-identified single participant anatomical and functional data, as well as group activation and connectivity contrast maps are available from the corresponding author upon reasonable request.

Human research participants

Policy information about studies involving human research participants and Sex and Gender in Research.

Reporting on sex and gender	Forty-seven healthy control participants were recruited for the study (25 male, 22 female; mean age, 24.0±0.5 years [± SEM]; range 19–37 years)
Population characteristics	See above
Recruitment	Participants were recruited through experimental notices distributed throughout the university of Melbourne, Australia. Due to the deceptive nature of the experiment (placebo analgesia), they were informed this study was investigating "human brain imaging of pain", looking at neural responses after application of a neutral control and active analgesic cream.
Ethics oversight	All experimental procedures were approved by the University of Sydney Human Research Ethics Committee and were consistent with the Declaration of Helsinki.

Note that full information on the approval of the study protocol must also be provided in the manuscript.

Field-specific reporting

Please select the one below that is the best fit for your research. If you are not sure, read the appropriate sections before making your selection.

☐ Life sciences ☒ Behavioural & social sciences ☐ Ecological, evolutionary & environmental sciences

For a reference copy of the document with all sections, see nature.com/documents/nr-reporting-summary-flat.pdf

Behavioural & social sciences study design

All studies must disclose on these points even when the disclosure is negative.

Study description	This study involved conditioning healthy human participants to believe a sham placebo cream labelled and described as "lidocaine", a potent analgesic, was working to modulate their pain relative to a control vaseline cream. Using human brain imaging, connectivity, and dynamic causal modelling, we hypothesise the existence of two seperate top-down networks which coordinate the output in descending modulatory pathways of the brainstem responsible for the manifestation of Placebo Analgesia.
Research sample	The research sample was largely formed by University students and researchers in adjacent fields at the University of Melbourne.
Sampling strategy	An apriori power analysis was conducted using Eippert et al. (2009) findings of cortico-brainstem communication. This revealed a total sample size of at least 40 would be necessary to detect similar effect sizes with 95% power ($d = 0.31$, $\alpha = 0.05$, power = 0.95). We elected to sample a larger number of participants due to signal artefact which can arise during human brain imaging, excluding some functional datasets. We observed no significant signal or structural artefact in any of our 47 participants, and as such included all functional data to meet the criteria of our power analysis and bolster the strength of any potential findings.
Data collection	Human brain imaging was recorded using the 7T MRI described above. Noxious stmuli were applied using a 3x3cm Peltier element thermode (Medoc). Pain rating data was recorded throughout the course of the study using a Visual Analogue Scale, which participants used to dynamically report their pain both outside (conditioning and reinforcement) and inside (test) the scanner.
Timing	Data collection occurred consistently throughout the years 2021-2022. Due to the constraints induced by COVID-19, we were unable to collect any data throughout lockdown periods in Australia.
Data exclusions	No data was excluded from this study
Non-participation	No participant drop out occurred throughout this study.
Randomization	Participants were allocated to a placebo "responder" or "nonresponder" group using the 2 Standard Deviation band method for determining significant deviations in typical pain processing that occur during placebo analgesia. This method involves calculating the standard deviation of pain responses in a single participant across a multi-trial acute pain design (stimulation of the control site), multiplying this number by two, and then calculating the average pain response in a single participant across a multi-trial modulated pain site (stimulation of the placebo "lidocaine" site). If this average is greater than two standard deviations lower than their pain responses on the control site, the participant is considered a responder. If they do not meet this criteria, they are considered a nonresponder.

Reporting for specific materials, systems and methods

We require information from authors about some types of materials, experimental systems and methods used in many studies. Here, indicate whether each material, system or method listed is relevant to your study. If you are not sure if a list item applies to your research, read the appropriate section before selecting a response.

Materials & experimental systems

n/a	Involved in the study
☒	☐ Antibodies
☒	☐ Eukaryotic cell lines
☒	☐ Palaeontology and archaeology
☒	☐ Animals and other organisms
☒	☐ Clinical data
☒	☐ Dual use research of concern

Methods

n/a	Involved in the study
☒	☐ ChIP-seq
☒	☐ Flow cytometry
☐	☒ MRI-based neuroimaging

Magnetic resonance imaging

Experimental design

Design type: Event-related design

Design specifications: Eight acute noxious stimuli were applied across the course of each functional sequence. Each stimulus lasted a total of 15 seconds, including a 4 degree / second ramp up from and ramp down to baseline.

Behavioral performance measures: Ongoing pain responses were recorded throughout all experimental phases using a computerized Visual Analogue Scale (VAS). That is, this rating system dynamically recorded participant pain responses at all times on a time scale consistent with volumes recorded during fMRI collection.

Acquisition

Imaging type(s): Structural (T1-weighted), and functional

Field strength: 7 Tesla

Sequence & imaging parameters: A T1-weighted anatomical image set covering the whole brain was collected (repetition time=5000 ms, echo time=3.1ms, raw voxel size=0.73x0.73x0.73mm, 224 sagittal slices, scan time=7mins). The two fMRI acquisitions each consisted of a series of 134 gradient echo echo-planar measurements using blood oxygen level dependant (BOLD) contrast covering the entire brain. Images were acquired in an interleaved collection pattern with a multi-band factor of four and an acceleration factor of three (repetition time=2500ms, echo time=26ms; raw voxel size=1.0x1.0x1.2mm, 124 axial slices, scan time=5:35mins).

Area of acquisition: Whole brain coverage was recorded in both the T1-weighted and Functional brain scans

Diffusion MRI: ☐ Used ☒ Not used

Preprocessing

Preprocessing software: Statistical Parametric Mapping Version 12 (SPM12). The first five volumes of each scan were removed from the model due to excessive signal saturation from the scanner. The remaining 129 functional images were slice-time and motion corrected and the resulting 6 directional movement parameters were inspected to ensure that all fMRI scans had no greater than 1mm of linear movement or 0.5 degrees of rotation movement in any direction. Images were spatially smoothed using a 6mm Full-width-at-half-maximum gaussian kernel.

Normalization: Each individual's fMRI image sets were then coregistered to their own T1-weighted anatomical, the T1 was then spatially normalized to the DARTEL template in Montreal Neurological Institute (MNI) space and the parameters applied to the fMRI image sets.

Normalization template: Dartel template in MNI space

Noise and artifact removal: Images were then linearly detrended to remove global signal changes, physiological noise relating to cardiac and respiratory frequency was removed using the DRIFTER toolbox (Särkkä, S. et al. NeuroImage 60, 1517-1527, (2012)), and the 6-parameter movement related signal changes were modelled and removed using a linear modelling of realignment parameters (LMRP) procedure.

Volume censoring: The first five volumes of each scan were removed from the model due to excessive signal saturation from the scanner.

Statistical modeling & inference

Model type and settings: Random-effects analyses, paired statistical analyses, Dynamic Causal Modelling settings: slice timing = 1.25s (modelled to the centre slice of acquisition), echo time = 0.026s, bilinear modulatory effects, one state per region, stochastic effects off, centred inputs on, and a timeseries fit. Mediation settings: FDR-corrected p<0.05, bootstrapped to 10,000 samples.

Effect(s) tested

Task-related connectivity was assessed using Psychophysiological Interaction Analysis (PPI). Task-independent connectivity was assessed using Functional Connectivity (FC). Directionality and effect size of connectivity differences assessed using Dynamic Causal Modelling (DCM). Mediation was used to test dependencies of connectivity relating to placebo responsivity and brainstem output (signal intensity change).

Specify type of analysis: ☐ Whole brain ☐ ROI-based ☒ Both

Anatomical location(s)

Brainstem-specific contrast maps using the SUIT toolbox were generated to identify the region within the Periaqueductal Gray matter (PAG) showing the greatest placebo-related difference in signal intensity between responder and nonresponder groups. A 1mm sphere centred on the peak-activated voxel was used in subsequent voxel-by-voxel whole brain connectivity analyses

Statistic type for inference
(See Eklund et al. 2016)

voxel-wise

Correction

p<0.001 uncorrected with 20 contiguous voxels were used for initial connectivity analyses. Significant voxels were small-volume corrected to reduce the likelihood of Type II errors. DCM was thresholded at connections with posterior probability >0.99, and mediation was performed at an FDR corrected threshold of p<0.05, bootstrapped to 10,000 samples.

Models & analysis

n/a	Involved in the study	
☐	☒	Functional and/or effective connectivity
☒	☐	Graph analysis
☐	☒	Multivariate modeling or predictive analysis

Functional and/or effective connectivity

Functional connectivity was conducted by how voxels of the brain correlated in signal change over time with the seed PAG region. Psychophysiological Interaction was conducted by convolving the seed timeseries with model timing (time points of stimulus delivery), and comparing how voxels in the brain correlated over time with the convolved series.

Multivariate modeling and predictive analysis

For Dynamic Causal Modelling analyses, posterior probabilities of the reduced model after nested search were thresholded at p>0.99, and effect sizes of parameter estimate differences between responders and non-responders were discerned using Cohen's d tests. Mediation analysis were performed between placebo responses and PAG seed signal intensity change values with connectivity estimates between cortical regions and the PAG seed entered as potential mediators. This analysis was performed at a false discovery rate correction of p<0.05, bootstrapped to 10,000 samples.
