# Supplementary material for: Stimulus-independent and stimulus-dependent neural networks underpin placebo analgesia responsiveness in humans
Source: Commun Biol. 2023 May 27;6:569. doi: 10.1038/s42003-023-04951-7 (PMC10224990; doi:10.1038/s42003-023-04951-7)
Supplement: Supplementary file 1 — Peer Review File [file 42003_2023_4951_MOESM1_ESM.pdf]

nature portfolio

Peer Review File

Stimulus-independent and stimulus-dependent neural networks underpin placebo analgesia responsiveness in humans

Open Access This file is licensed under a Creative Commons Attribution 4.0 International License, which permits use, sharing, adaptation, distribution and reproduction in any medium or format, as long as you give appropriate credit to the original author(s) and the source, provide a link to the Creative Commons license, and indicate if changes were made. In the cases where the authors are anonymous, such as is the case for the reports of anonymous peer reviewers, author attribution should be to 'Anonymous Referee' followed by a clear attribution to the source work. The images or other third party material in this file are included in the article's Creative Commons license, unless indicated otherwise in a credit line to the material. If material is not included in the article's Creative Commons license and your intended use is not permitted by statutory regulation or exceeds the permitted use, you will need to obtain permission directly from the copyright holder. To view a copy of this license, visit http://creativecommons.org/licenses/by/4.0/.

Reviewers' comments:

Reviewer #1 (Remarks to the Author):

In this manuscript Crawford et al. investigate the neural networks underlying placebo analgesia capitalizing on the strength of 7T high-field MRI. The authors focus on placebo-related connectivity of the PAG and report two independent networks to contribute to placebo analgesia, i.e. one stimulus-dependent and one stimulus-independent network.
Generally, this is a very interesting and methodologically sophisticated approach and the results would provide an important advance in our understanding of the distinct mechanisms – particularly those involving the brainstem – underlying placebo analgesia.

However, I have several comments that need to be addressed or clarified before the validity of the results can be fully evaluated.

Major comments:
• The authors use the term "stimulus independent" and "stimulus-dependent" network. As far as I understand the stimulus-dependent network is identified via a PPI that involves the pain-stimulus regressor, which makes sense. However, the proposed "stimulus independent" network is determined by assessing any changes in connectivity occurring over the scanning session (including stimulus presentation). Please clarify.
• I also wonder how the typical use of temporal filtering in task-related fMRI interferes with the assessment of the tonic connectivity changes investigated here.
• In analysis 1 and 2 FC and PPI were used to identify regions which are associated with placebo related activity in the PAG. The authors interpreted their results as the placebo responders showed altered connection, however, the statistical test to support that is not reported. The identified region showed a significant "main effect" between placebo and control sessions, but not between responder and non-responders.
• Along these lines I wonder why the dichotomous approach of comparing responders with non-responders was used at all. Why not include an individual's response as a continuous variable which has much more statistical power?
• The authors should at least discuss why no placebo effect was observed in this sample, although participants were conditioned twice.
Further comments and methodological aspects that require a more detailed description.
• In the main text the authors refer to a VAS 10 but in the methods part and on the images they show VAS 100.
• All statistical differences should be reported with its results(t/F value) eg :behavioural results, PAG activation, etc.
• The rating procedure remains unclear. The authors report that participants "continuously rated their pain experience". Did participant rate after each stimulus (ie:within rating period) or during the stimulus itself? If they continuously rated during the stimulus (or even during the whole scanning session?), which values were actually taken to the analysis?

• on page 17 , last row: "These three temperatures.." The authors only mention two temperatures.
• Please report the used temperatures on a group level in the supplementary.
• Methods: Reinforcement and Test part: "creams were applied to the volar surface of both left and right forearms" - however, the authors only mention the right forearm in the description of session 1.
• Please describe the design in more detail:
o Was the expectancy measured once before each run (set of stimuli) or before each stimulus?
o In the design and following design matrix: did the authors use an anticipation phase? Or rating

intervals ?
• The authors report a total scan time of 6:25 per session. However, the reported number of volumes and TR does not reflect it(TR(2.5s)*Nvol(134)=5:35). Additionally on Figure 2C, the design visualization suggests that a rest period was used at the beginning of each session. Please specify.
• The motion parameters were checked, but no information is provided about how many volunteers had higher values than the set threshold.
• Please specify in the method part that cardiac and respiratory signal was measured (now it is only in the preprocessing part).
• The authors sometimes refer to the two sessions as control and placebo and sometimes as vaseline and lidocain. Please harmonize.

Reviewer #2 (Remarks to the Author):

This is an innovative and interesting study on placebo responsiveness and functional connectivity used to differentiate placebo responders from nonresponders.
The authors separate the role of the lPAG into a stimulus-dependent and non-stimulus independent network. Rationale for linking the stimulus-independent to the hypothamus and the stimulus-independent to the rACC need to be emphasized. A stronger rational for the network being investigated should be added.

The classification of individuals as responder (n=23) or nonresponder (n=24) participants using the two-standard deviation band method has been criticized by some. Other approaches like the permutation test have been suggested and the authors may want to apply it and re-run the analyses.

A stronger justification for using DCM and more explicitly outlining the conclusions that can be drawn, is needed.

For the mediation model, explain how the PPI of lPAG-rACC and lPAG-PH are set as mediators of an outcome of lPAG activation. It seems like they're too similar of metrics to be used as a mediator AND the outcome.

Overall analyses with the entire sample size shall be included for the PPI and FC analyses.
The conclusion about alteration of the network in chronic conditions should be revised and updated with the published studies on placebo and chronic pain.

A figure with each single subject can be added.

Biology Comms Reviewer Comments

Reviewer #1 (Remarks to the Author):

1) The authors use the term "stimulus independent" and "stimulus-dependent" network. As far as I understand the stimulus-dependent network is identified via a PPI that involves the pain-stimulus regressor, which makes sense. However, the proposed "stimulus independent" network is determined by assessing any changes in connectivity occurring over the scanning session (including stimulus presentation). Please clarify.

This is correct – the *stimulus-independent* network was identified by running a design to reveal any voxel in the brain that either correlated or anticorrelated in BOLD fluctuations within the PAG seed across the entire scan period. This includes the 90 second baseline, 8 noxious stimuli, and their associated interstimulus intervals. The plateau period of each stimulus (where stimulus presentation has reached noxious intensity) occupied roughly 7.5-9 seconds of the stimulus presentation, relating to roughly 21% of the total scan period. Thus, whilst the changes in PAG coupling identified in the stimulus-independent network include the 21% of scan time where noxious periods were applied, they also include the 79% of scan time where they weren't – and these same periods are regressed out of the stimulus-dependent network by the generation of the interaction term. These two connectivity analysis paradigms reveal separate neural associations with the PAG – those which occur strictly during noxious stimuli and those that occur both outside and during the time windows where noxious stimuli are presented, yet still within the context of expecting pain relief.

We have altered the description of the *stimulus independent-network* analysis to clarify how this regressor is generated.

Page 22:

"stimulus independent connectivity changes in responders and non-responders were assessed by conducting a functional connectivity (FC) analysis. This analysis generates contrast images with includes the timeseries of the PAG seed as a regressor, independent to the timing of noxious stimuli applied (Fig 2A). As such, this analysis reveals cortical regions contacting the PAG during the entire scan period including the baseline anticipation, pain, ramp, and inter-stimulus-interval periods ~~inter-stimuli periods~~. Using these contrast images, a random-effects paired, voxel-by-voxel analysis was conducted in placebo responders comparing the control vaseline- and placebo lidocaine-site series ~~(Figure 2B, C)~~. From resulting clusters, eigenvariates representing stimulus-independent connectivity with the PAG in each series were extracted from both placebo responder and non-responder contrast images and significance was determined in both groups using paired t-tests (Figure 2B,C)."

2) I also wonder how the typical use of temporal filtering in task-related fMRI interferes with the assessment of the tonic connectivity changes investigated here.

The only temporal filtering applied to our fMRI data consisted of filtering respiratory and cardiac signal (via the Drifter toolbox), as well as global intensity normalization via

a linear detrending function. These steps were important for identifying signal intensity change specifically relating to noxious stimulus application – which was integral to first establishing where in the PAG the greatest change in activation occurred, establishing a seed for subsequent connectivity analyses. Secondly, these steps were important to reduce scan-to-scan variability relating to signal drift and global signal intensity.

We propose that these temporal filtering strategies would not have interfered with the tonic connectivity differences between groups we identified in the stimulus independent network as: i) both responders and non-responder contrast maps were subject to the same temporal filtering steps; ii) the regions we identify as displaying altered tonic coupling with the PAG in placebo responders have all previously been identified as playing a role in regulating the sensitivity of brainstem pathways involved in behavioural responses and analgesia (Huang & Flynn, 1975; Ongür et al., 1998), giving us a strong explanation for both their presence as well as how their altered coupling with the PAG may be involved in mounting endogenous pain modulatory responses.

3) In analysis 1 and 2 FC and PPI were used to identify regions which are associated with placebo related activity in the PAG. The authors interpreted their results as the placebo responders showed altered connection, however, the statistical test to support that is not reported. The identified region showed a significant "main effect" between placebo and control sessions, but not between responder and non-responders.

Each cluster revealed through the FC and PPI analysis showed a "main effect" of placebo in the placebo responder group only, and no significant change in connectivity in the non-responder group. These measures of significance were generated through paired t-tests, as detailed on page 22.

For example – taken from the bar plots in figure 2 – responders show a placebo main effect and no change in PAG coupling is found in non-responders within these same significant clusters.

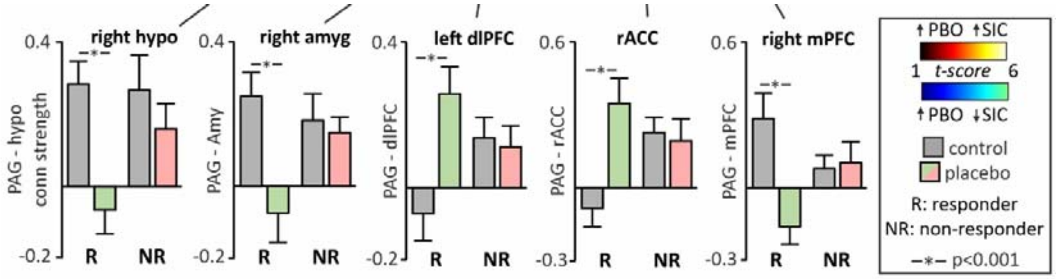

We should have made this analysis step clearer and have now clarified the wording in the methods section to reflect how these analyses were performed:

Page 22:

"Using these contrast images, a random-effects paired, voxel-by-voxel analysis was conducted in placebo responders comparing the control vaseline- and placebo lidocaine-site scans ~~vaseline and lidocaine stimulated series~~. From resulting clusters, eigenvariates representing stimulus independent PAG connectivity in each series

were extracted from both placebo responder and non-responder contrast images and significance was determined in both groups using paired t-tests (Figure 2E, F)."

Page 23:

"Using these contrast images, a random-effects paired, voxel-by-voxel analysis was conducted in placebo responders comparing the control vaseline- and placebo lidocaine-site series ~~(Figure 2B, C)~~. From resulting clusters, eigenvariates representing stimulus dependent connectivity with the PAG in each series were extracted from both placebo responder and non-responder contrast images and significance was determined in both groups using paired t-tests (Figure 2B,C)."

4) Along these lines I wonder why the dichotomous approach of comparing responders with non-responders was used at all. Why not include an individual's response as a continuous variable which has much more statistical power?

The dichotomous approach of separating individuals into responders and non-responders is used regularly by many placebo analgesia studies (Benedetti, 1996; Levine et al., 1979; Zubieta et al., 2005), and we have used this method in the past to reveal neural differences between those that do and do not mount various endogenous pain modulatory responses (Crawford et al., 2021; Youssef et al., 2016). The major limitation with using a continuous variable is that it does not account for individual variability in baseline pain intensity perceptions. Whilst rarely reported, individuals given the same noxious stimulus intensity can rate the intensity of pain with considerable variability. We have recently published a manuscript detailing this phenomenon (Crawford et al., 2022). Given this, it is possible that a decrease of for example 10 on a VAS (0-100) could be a significant decrease in a non-variable baseline individual but not significant in a variable baseline individual.

In addition, using a continuous variable approach, clusters demonstrating a linear change in say PAG connectivity with perceived pain would also include those individuals in which pain *increased* when the placebo lidocaine-site was stimulated relative to the control vaseline-site. Whilst we agree that there is validity in exploring the magnitude of analgesia against brain function, we suggest that our interpretation of the placebo analgesia phenomena as either being expressed or not expressed in a proportion of individuals is the best approach. Our findings of two separate brain networks which regulate and drive brainstem analgesic output in placebo responders and not in non-responders suggests that the dichotomous approach is of value.

We have now added a limitations paragraph to the manuscript highlighting that we do perform a dichotomous responder versus non-responder approach, and that there exists dispute in the field over how best to conduct these analyses.

"Although we utilized PPI, FC, and directed connectivity analyses to unveil the most integral cortical networks that regulate and drive PAG output during placebo analgesia – these networks were identified by considering the phenomena as dichotomous – that is, we delineated and investigated a responder and non-responder group. Performing these analyses allowed us first to identify which cortical connections with

the PAG were significantly altered in those demonstrating a placebo response as determined through the 2SD band method, and then assess these connections in non-responders for statistical differences. There exists conflicting literature over the method of determining placebo responses (for example by using arbitrary VAS changes or permutation testing), as well as if placebo analgesia should be considered a continuous variable (Jensen et al., 2015; Kaptchuk et al., 2008; Olsen et al., 2018). Whilst these approaches are well- documented, the former is limited by not considering an individual's perceived pain intensity baseline variability, and the latter would only identify clusters where PAG connectivity shows a linear relationship with graded changes in perceived pain – including in those where pain either did not change between the control vaseline- and placebo lidocaine-sites or indeed in those who's pain increased when exposed to placebo. As such, in our analysis we can be confident that we have described a functional architecture underpinning significant placebo analgesia, and that these same connections are unchanged in non-responders."

5) The authors should at least discuss why no placebo effect was observed in this sample, although participants were conditioned twice.

The response rate we observed in our participant population (48%) is well-aligned to previous investigations using response conditioning models to elicit placebo analgesia (Petrovic et al., 2002), and it is well understood that not all individuals demonstrate the phenomenon. Indeed, several genetic and neurobiological markers have been proposed for why there is such inter-individual variability in placebo analgesia expression, however our investigation lacks the required analytic techniques to assess if these are the principal underlying factors "encoding" a placebo non-responder. Indeed, we have a planned investigation commencing this year to assess the consistency of placebo responses with a rich dataset of genetic and psychological components, which we hope will shed light on whether there exists a combination of traits and biological components which can predict who will show a placebo response and who will not over time.

That being said, we have added to our limitations section a future direction which discusses this question, which we hope will prompt future study into a better understanding of the variable nature of this phenomenon.

"It is of note that despite receiving an identical response conditioning protocol, over half our sample did not demonstrate a significant placebo response. Whilst the focus of this investigation was to identify the functional networks of placebo responders, it would also be of interest to better understand the driving factors influencing why certain individuals fail to generate significant analgesic responses to placebo. Two leading theories – the Bayesian brain hypothesis (Pagnini et al., 2023) and an individual's underlying biological substrates (Scott et al., 2008) both centre on error-prediction signalling and dopaminergic neurotransmission, which encompass roles of the NAc. Since we identified this region as feeding into the rACC-PAG pathway in a stimulus-dependent manner, it may be that this cortical site is a key delineating factor between an individual forming accurate stimulus-response relationships and generating placebo responses via response conditioning. Future studies could

compliment this work by assessing the role of the NAc in the conditioning phases of placebo analgesia, and indeed whether NAc activation or neurotransmission during these phases could be a potential biomarker of placebo responsiveness."

Further comments and methodological aspects that require a more detailed description.

6) In the main text the authors refer to a VAS 10 but, in the methods part, and on the images they show VAS 100.

This has now been amended on page 4 of the manuscript body – indeed the rating scale extended from 0 (no pain at all) to 100 (worst pain imaginable), and is now consistent throughout the body and methods sections.

7) All statistical differences should be reported with its results (t/F value) eg: behavioural results, PAG activation, etc.

We have now included significance values (p-values for 2-sample and paired tests, f-value for ANOVA) with their associated behavioural and activation tests reported within the manuscript.

Page 5:

"Despite both groups expecting reduced pain on the placebo lidocaine-treated site (mean±SEM expectation responder: vaseline = 49.3±0.8, lidocaine = 33.5±1.6, $p < 0.001$; non-responder: vaseline = 51.7±1.8, lidocaine = 37.1±1.6, $p < 0.001$), only 23 of the 47 participants demonstrated a significant pain reduction when identical intensity stimuli were applied to both sites (mean±SEM VAS responder: vaseline = 45.2±1.5, lidocaine = 32.9±1.9, $p < 0.001$; non-responder: vaseline = 42.2±2.8, lidocaine = 45.9±2.4, $p = 0.09$) (Fig 1C). Pain rating responses to the control Vaseline-site did not differ between response and non-responder groups ($F_{2,46}$ = 2.59, p = 0.22). Additionally, inspection of the low and moderate temperatures applied throughout conditioning and test phases revealed no differences between placebo responder and non-responder groups (Supplementary table 1). Group-level analyses of placebo responder and non-responder groups revealed a significant and differential engagement of the lPAG, consistent with a previous report (mean±SEM change in β value responder: -0.56±0.33; non-responder: 1.15±0.24; $p < 0.001$) (Fig 1D)."

Supplementary Table 1:

Table 1: Temperatures applied throughout conditioning and test phases in placebo responder and nonresponder groups.

	Responder	Nonresponder	P-value
**Moderate Temperature (degrees±SEM)**	46.8±0.17	46.7±0.20	0.94
**Low Temperature (degrees±SEM)**	45.8±0.17	45.6±0.22	0.57

8) The rating procedure remains unclear. The authors report that participants "continuously rated their pain experience". Did participant rate after each stimulus (ie:within rating period) or during the stimulus itself? If they continuously rated during the stimulus (or even during the whole scanning session?), which values were actually taken to the analysis?

Participants were instructed to report their pain continuously throughout the scan. There was no explicit rating period modelled after each stimulus presentation, but rather on ongoing trace of pain intensity ratings were generated for each participant. Pain rating values were extracted for each 0.5 seconds and values during each pain period were averaged providing a pain intensity for each noxious stimulus period. We have amended the methods section to clarify this procedure.

Page 19 of the manuscript methods:

"VAS responses were recorded every 0.5 seconds, and values during each pain period were averaged providing a pain intensity for each noxious stimulus period."

9) on page 17 , last row: "These three temperatures.." The authors only mention two temperatures.

We have now altered this section of the manuscript – we only used two temperatures throughout conditioning, a low and moderate temperature.

10) Please report the used temperatures on a group level in the supplementary.

We have now created an additional supplementary table including this information. This table is now titled "Table 1" and each other supplementary table has been moved forward to accommodate this change.

Page 5:

"Inspection of the low and moderate temperatures applied throughout conditioning and test phases revealed no differences between placebo responder and non-responder groups (Supplementary table 1)."

Supplementary Table 1:

Table 1: Temperatures applied throughout conditioning and test phases in placebo responder and nonresponder groups.

	Responder	Nonresponder	P-value
**Moderate Temperature (degrees±SEM)**	46.8±0.17	46.7±0.20	0.94
**Low Temperature (degrees±SEM)**	45.8±0.17	45.6±0.22	0.57

11) Methods: Reinforcement and Test part: "creams were applied to the volar surface of both left and right forearms" - however, the authors only mention the right forearm in the description of session 1.

This is correct, during session 1 the creams were only applied to the right forearm, as this is the same surface stimulated during test. The rationale for applying the creams to the surfaces of both forearms during "Methods: Reinforcement and Test" is that due to the close temporal spacing between the reinforcement and test phases, we wanted to ensure that no lingering sensitivity induced through reinforcement remained on the test surface (right forearm). Therefore, reinforcement was conducted on the left forearm, after which testing is performed on the right forearm, in the same locations as conditioning to create as similar an environment as possible to when participants were undergoing response conditioning.

We have now clarified this point in the "Reinforcement and Test" section of the methods:

"To ensure that the protocol for conditioning was consistent between subsequent days, and the change in immediate environment (inside the MRI), reinforcement was conducted by applying four noxious stimuli at the same low and moderate temperatures used throughout session 1 to participants' left volar forearm. This was performed on the opposite forearm to prevent sensitization of the testing area (the right volar forearm)."

Please describe the design in more detail:

12) Was the expectancy measured once before each run (set of stimuli) or before each stimulus?

Yes, expectancy was measured once before each run – this has now been clarified in the methods section page 19 of the manuscript.

"We asked each participant for an average expectation of pain intensity directly prior to each stimulation series and instructed them to report the pain intensity continuously throughout ~~experienced over~~ the duration of the scan using the button box and the projected digital VAS."

13) in the design and following design matrix: did the authors use an anticipation phase? Or rating intervals?

The design matrix included volumes of the scan corresponding to the plateau period of each noxious stimulus. No anticipation phases were included or modelled in the subsequent connectivity analyses.

We have now clarified the boxcar design and subsequent design matrix (as this was used for both the initial PAG activation and PPI regressor analyses) and the inclusion of the 90-second baseline period.

Page 19:

"Each participant received two consecutive series of eight stimuli, with a separate functional series collected during each series of stimuli. Each fMRI series began with a 90-second baseline period prior to the eight stimuli presentations."

Page 22:

"To determine significant changes in signal intensity during each noxious thermal period, a repeating boxcar model convolved with a canonical hemodynamic response function was applied to each of the fMRI series. Within this model, scanning volumes overlying stimulus plateau periods were assigned a value of 1, and inter-stimulus-intervals and the initial 90-second baseline period were assigned a value of 0."

*14) The authors report a total scan time of 6:25 per session. However, the reported number of volumes and TR does not reflect it(TR(2.5s)*Nvol(134)=5:35). Additionally on Figure 2C, the design visualization suggests that a rest period was used at the beginning of each session. Please specify.*

Our apologies – this is an oversight which has now been amended, indeed the actual collection time of functional volumes is 5min 35sec - we amended the manuscript accordingly

In regard to the rest period preceding stimulus application. We include a 90 second period at the beginning of each functional scan to include a greater number of "0" volumes in the boxcar model to assess changes in neural activation during noxious stimuli. Its inclusion improves our ability to assess the baseline function of an individual's brain outside of painful periods. We have now amended the manuscript explaining its inclusion:

Page 19:

"Each participant received two consecutive series of eight stimuli, with a separate functional series collected during each series of stimuli. Each fMRI series began with a 90-second baseline period prior to the eight stimuli presentations."

Page 22:

"To determine significant changes in signal intensity during each noxious thermal period, a repeating boxcar model convolved with a canonical hemodynamic response function was applied to each of the fMRI series. Within this model, scanning volumes overlying stimulus plateau periods were assigned a value of 1, and inter-stimulus-intervals and the initial 90-second baseline period were assigned a value of 0."

15) The motion parameters were checked, but no information is provided about how many volunteers had higher values than the set threshold.

In no participant did motion parameters exceed our elected threshold (1mm linear, 0.5 degrees rotational). This is not uncommon in our experience using the 7T scanner and their support sponges which both support the head yet restrict lateral and rotational motion. We have added this information to the methods section.

Page 20:

"resulting 6 directional movement parameters were inspected to ensure that all fMRI scans had no greater than 1mm of linear movement or 0.5 degrees of rotation movement in any direction. In no single participant in either the placebo lidocaine- or control vaseline-site scans did motion parameters exceed our elected threshold."

16) Please specify in the method part that cardiac and respiratory signal was measured (now it is only in the preprocessing part).

Cardiac and respiratory signal was not directly measured for this investigation but rather modelled and removed from the data using the frequency band of simulated data within the range most commonly expressed by healthy individuals within the scanner environment (Cardiac = 60-120 beats per minute; respiratory = 8-25 breaths per minute). This information has now been included within the methods section to clarify this point.

"Images were then linearly detrended to remove global signal changes, physiological noise relating to cardiac (frequency band of 60-120 beats per minute +1 harmonic) and respiratory (frequency band of 8-25 breaths per minute +1 harmonic) frequency was removed using the DRIFTER toolbox (Särkkä et al., 2012),"

17) The authors sometimes refer to the two sessions as control and placebo and sometimes as vaseline and lidocain. Please harmonize.

This has now been harmonized, and in all instances of describing the two creams, we have elected to use "control vaseline-site" and "placebo lidocaine-site"

Reviewer #2 (Remarks to the Author):

1) The authors separate the role of the lPAG into a stimulus-dependent and non-stimulus independent network. Rationale for linking the stimulus-independent to the hypothamus and the stimulus-independent to the rACC need to be emphasized. A stronger rational for the network being investigated should be added.

We have now added additional rationale for our hypothesis in linking the hypothalamus-PAG connection to the stimulus-independent network and rACC-PAG connection to the stimulus-dependent network.

Page 3:

“Preclinical investigations have revealed that lPAG stimulation evokes emotional coping behaviours, of which analgesia is an integral component (Mokhtar & Singh, 2020). While the lPAG can produce these behaviours without input from higher centres, it was shown over half a century ago that the sensitivity of lPAG is tonically regulated by hypothalamic inputs (Eclancher & Schmitt, 1972; Ellison & Flynn, 1968; Huang & Flynn, 1975; Wang et al., 2021). In humans, the hypothalamus forms part of the lower pain control system and is involved in both pain control and maintaining autonomic homeostasis via its reciprocal connection with the lPAG (Craig, 2003; Stroman et al., 2018). Additionally, both hypothalamic and midbrain activation has been observed during placebo analgesia, suggesting a phylogenetically conserved system of pain control exists consisting of subcortical and brainstem structures including the hypothalamus and PAG (Eippert, Finsterbusch, et al., 2009).

Page 4:

“Importantly, placebo analgesic responses are not expressed in all individuals and what determines placebo analgesia responsivity remains unknown. Given the abovementioned preclinical and human data, it is possible that on-going modulation of the lPAG by the hypothalamus determines whether or not an individual expresses placebo analgesia. ~~Additionally, p~~Placebo analgesia is also associated with noxious stimulus-evoked activity changes in higher brain regions including the rostral anterior cingulate cortex (rACC) and dorsolateral prefrontal cortex (dlPFC) (Eippert, Bingel, et al., 2009; Wager et al., 2004). These activation patterns are accompanied by heightened μ-opioid binding in the rACC and coupling between the rACC and PAG (Scott et al., 2008; Wager et al., 2007), and the expression of placebo analgesia can be blocked by the administration of the opioid antagonist naloxone. When naloxone is administered, the attenuation of these responses is associated with a reduction in rACC-PAG connectivity (Eippert, Bingel, et al., 2009).

2) The classification of individuals as responder (n=23) or non-responder (n=24) participants using the two-standard deviation band method has been criticized by some. Other approaches like the permutation test have been suggested and the authors may want to apply it and re-run the analyses.

We agree that there is some criticism in the behavioural neuroscience community surrounding the use of the two standard deviation band method, and have in recent years found success in delineating placebo responders from non-responders to a variety of endogenous pain modulatory phenomena using this method (Crawford et al., 2021; Youssef et al., 2016). That being said, we have run a permutation test (10,000 permutations) on our psychometric pain rating data and only two of the 47 participants changed in their classification, moving from the non-responder to the responder group.

We would argue that our 2-standard deviation band method is slightly more stringent than the permutation testing and found that our imaging results remain unchanged

when these two participants were moved from the non-responder to responder group. In the future we will continue to cross reference our 2-standard deviation band method with permutation testing, and if we find a significant discrepancy will consider altering our method of group classification.

3) A stronger justification for using DCM and more explicitly outlining the conclusions that can be drawn, is needed.

We have now included additional information supporting the addition of DCM to assess directed connectivity between brain regions, as well as adjusted the scope of conclusions which can be drawn from DCM output data.

Page 5:

"Although it has long been proposed that top-down recruitment of analgesic brainstem pathways underpins placebo analgesia, information on directionality of seed-to-voxel relationships cannot be gleaned from these connectivity analyses alone (Necka et al., 2019). As such ~~Additionally~~, we additionally conducted Dynamic Causal Modelling (DCM) and a multiple mediation analysis ~~on resulting clusters~~ to determine directed connectivity between cortical and subcortical regions (i.e., if placebo analgesia was associated with top-down or bottom-up projections), as well as determine which regions were working either independently or as a system to drive the relationship between lPAG activity and placebo responses."

Page 11:

"Additionally, as we conducted our DCM and mediation analyses using clusters that were first revealed using the lPAG timeseries as a seed, our results are constrained to solely regions which likely receive information from or project directly to the lPAG. Whilst this does not allow assessment of regions which comprise alternate projection pathways which may be involved in the response, encoding more nuanced aspects of placebo analgesia such as cognitive evaluation or complex emotional processing, the results presented do offer valuable insight into the functional projections regulating and driving brainstem output in humans to produce an antinociceptive state. "

4) For the mediation model, explain how the PPI of lPAG-rACC and lPAG-PH are set as mediators of an outcome of lPAG activation. It seems like they're too similar of metrics to be used as a mediator AND the outcome.

Since we first used DCM to establish that our connectivity results indeed reflected a top-down modulation of the lPAG by the rACC / PH, we believe that the use of these connectivity weightings as mediators is appropriate. That is, at the time of performing mediation we already had a strong indication that the connectivity between these two sites and the lPAG played an important role in regulating the degree of signal intensity change observed within the lPAG. Mediation in this instance was used solely to establish how the coupling between the rACC, PH, and lPAG interacted with the established relationship between placebo responsiveness and lPAG activation (Figure

1). Whilst they are similar metrics, similar analyses have been performed in the past using the activation in one brain region as the mediator for activation in a second brain region, and similarly, that the connectivity between two brain regions mediates two behavioural variables (Schenk & Colloca, 2019; Wager et al., 2008). Where our analysis differs is that we instead propose that top-down functional coupling from a connected voxel to a seed can influence the timeseries of the initial seed.

In our investigation, mediation is used to bolster the findings first established in DCM – that is, as an individual's coupling between the rACC and PAG increases, so too does their activation within the PAG and this interaction relates to the likelihood of an individual showing a significant placebo response. Controlling for the coupling between the rACC-PAG abolishes the relationship we establish in Figure 1 between placebo responses and PAG activation (as the a*b path effect is significant and c' is not). In contrast, whilst PH-PAG coupling relates to placebo responder group classification, it does not directly influence activation of the lPAG. This is in-line with the DCM results as well, as these nodes appear to influence rather the lPAG-lPAG self-connection (Friston et al., 2003), establishing the excitatory tone of the lPAG to be influenced by other extrinsic connections (which we propose is the top down modulation of the lPAG by the rACC which demonstrates complete mediation).

5) Overall analyses with the entire sample size shall be included for the PPI and FC analyses.

The aim of the present investigation was first to identify which regions in individuals demonstrating a significant placebo response demonstrated connectivity changes with the PAG in either a stimulus-dependent (PPI) or -independent (FC) manner, and then assess if these connections were similarly altered in those who did not display the phenomena. As such, inclusion of a main effect analysis as described by the reviewer would dilute the effect size observed in responders, or indeed hide the significant connections we identify. In addition, it would prevent the ability of any statistical analysis being performed on subgroups (high and low placebo responders) by the tenets of circularity. We have however added to our limitations section describing that we have performed this dichotomous analysis, with the rationale for doing so and the limitations of considering VAS change as a continuous variable or conducting main effect analyses specifically when looking at placebo analgesia.

"Although we utilized PPI, FC, and directed connectivity analyses to unveil the most integral cortical networks that regulate and drive PAG output during placebo analgesia – these networks were identified by considering the phenomena as dichotomous. That is, we delineated and investigated a responder and non-responder group. Performing these analyses allowed us first to identify which cortical connections with the PAG were significantly altered in those demonstrating a placebo response as determined through the 2SD band method, and then assess these connections in non-responders for statistical differences. There exists conflicting literature over the method of determining placebo responses (for example by using arbitrary VAS changes or permutation testing), as well as if placebo analgesia should be considered a continuous variable (Jensen et al., 2015; Kaptchuk et al., 2008; Olsen et al., 2018).

Whilst these approaches are well- documented, the former is limited by not considering an individual's perceived pain intensity baseline variability, and the latter would only identify clusters where PAG connectivity shows a linear relationship with graded changes in perceived pain – including in those where pain either did not change between the control vaseline- and placebo lidocaine-sites or indeed in those who's pain increased when exposed to placebo. As such, in our analysis we can be confident that we have described a functional architecture underpinning significant placebo analgesia, and that these same connections are unchanged in non-responders.“

6) The conclusion about alteration of the network in chronic conditions should be revised and updated with the published studies on placebo and chronic pain.

We have now expanded our concluding remarks to include a selection of up to date references in placebo and chronic pain.

"In conclusion, we provide evidence for two brain networks responsible for altering descending brainstem pathways during placebo analgesia. Whilst this investigation utilized a specific protocol consisting of short lasting thermal stimuli and response conditioning ~~deceptive creams~~ to induce analgesic effects, regions in the network we describe have previously been tied with analgesia elicited from longer lasting stimuli or chronic conditions (Eippert, Bingel, et al., 2009; Schmid et al., 2015). Recently, brainstem projections from the hypothalamus have been linked to pain anticipation in Fibromyalgia patients (Ioachim et al., 2022), and the cingulate cortex – specifically it's anterior division - has been proposed as a neurosurgical target to treat intractable pain due to its role in emotional and attentional processing during painful events (Peyron et al., 2019). ~~as well as in response~~ Additionally, the same regions we identify from placebo analgesia generated by response conditioning appear to be involved in alternative placebo substances such as social observation which includes the amygdala and PAG (Schenk & Colloca, 2019), and pharmacological conditioning which include the rACC and PAG ~~medication~~ (Vachon-Presseau et al., 2018). These data are consistent with frontotemporal and limbic structures playing a generalized role in recruiting brainstem pain-modulatory circuits to drive analgesia, emphasising the role of "mind set" and emotion in influencing our responses to pain. Indeed, it remains to be seen whether these specific connections between cortical, subcortical, and brainstem sites ~~cortico-brainstem pain control mechanisms~~ are compromised in individuals with chronic pain or underpin alternative endogenous pain modulatory phenomena."

7) A figure with each single subject can be added.

We have now demographic information for each participant included in this investigation (age, gender, etc.). This information has now been included in the supplement as supplementary table 6.

Table 6. Demographic data of each participant's age, gender, and group assignment for placebo responders and non-responders.

Responders n=23			Non-responders n=24		
Subject ID	Age	Gender	Subject ID	Age	Gender
2013	20	M	2009	20	M
2015	19	F	2010	20	F
2022	20	F	2011	20	M
2025	22	F	2014	21	M
2028	24	F	2016	19	F
2035	24	F	2021	24	F
2036	21	M	2023	22	M
2037	21	M	2024	21	M
2039	32	F	2026	33	M
2049	23	M	2027	24	M
2056	24	M	2029	27	F
2078	24	F	2030	23	F
2079	25	M	2032	20	F
2082	29	M	2034	23	F
2083	21	F	2040	26	M
2084	25	M	2041	22	M
2085	24	M	2077	23	F
2086	32	F	2080	24	F
2087	25	M	2081	31	M
2088	25	M	2091	23	M
2089	25	F	2095	27	F
2090	24	M	2097	23	F
2094	23	M	2098	23	M
			2099	37	F
Mean±SEM	24.0±0.68	13M:10F		24.0±0.84	12M:12F

References:

Benedetti, F. (1996). The opposite effects of the opiate antagonist naloxone and the cholecystokinin antagonist proglumide on placebo analgesia. *Pain, 64*(3), 535-543. doi:https://doi.org/10.1016/0304-3959(95)00179-4

Craig, A. D. (2003). Interoception: the sense of the physiological condition of the body. *Curr Opin Neurobiol, 13*(4), 500-505. doi:10.1016/s0959-4388(03)00090-4

Crawford, L., Mills, E., Meylakh, N., Macey, P. M., Macefield, V. G., & Henderson, L. A. (2022). Brain activity changes associated with pain perception variability. *Cerebral Cortex*. doi:10.1093/cercor/bhac332

Crawford, L. S., Mills, E. P., Hanson, T., Macey, P. M., Glarin, R., Macefield, V. G., . . . Henderson, L. A. (2021). Brainstem Mechanisms of Pain Modulation: A within-Subjects 7T fMRI Study of Placebo Analgesic and Nocebo Hyperalgesic Responses. *The Journal of Neuroscience, 41*(47), 9794-9806. doi:10.1523/jneurosci.0806-21.2021

Eclancher, F., & Schmitt, P. (1972). [Effect of early lesions of the amygdala and median hypothalamus on the development of interspecific aggression behavior in rats]. *J Physiol (Paris), 65*, Suppl:231A.

Eippert, F., Bingel, U., Schoell, E. D., Yacubian, J., Klinger, R., Lorenz, J., & Büchel, C. (2009). Activation of the Opioidergic Descending Pain Control System Underlies Placebo Analgesia. *Neuron, 63*(4), 533-543. doi:10.1016/j.neuron.2009.07.014

Eippert, F., Finsterbusch, J., Bingel, U., & Büchel, C. (2009). Direct evidence for spinal cord involvement in placebo analgesia. *Science, 326*(5951), 404. doi:10.1126/science.1180142

Ellison, G. D., & Flynn, J. P. (1968). Organized aggressive behavior in cats after surgical isolation of the hypothalamus. *Arch Ital Biol, 106*(1), 1-20.

Friston, K. J., Harrison, L., & Penny, W. (2003). Dynamic causal modelling. *NeuroImage, 19*(4), 1273-1302. doi:https://doi.org/10.1016/S1053-8119(03)00202-7

Huang, Y. H., & Flynn, J. P. (1975). Unit activities in the hypothalamus and midbrain during stimulation of hypothalamic attack sites. *Brain Res, 93*(3), 419-440. doi:10.1016/0006-8993(75)90181-x

Ioachim, G., Warren, H. J. M., Powers, J. M., Staud, R., Pukall, C. F., & Stroman, P. W. (2022). Altered Pain in the Brainstem and Spinal Cord of Fibromyalgia Patients During the Anticipation and Experience of Experimental Pain. *Front Neurol, 13*, 862976. doi:10.3389/fneur.2022.862976

Jensen, K. B., Kaptchuk, T. J., Chen, X., Kirsch, I., Ingvar, M., Gollub, R. L., & Kong, J. (2015). A Neural Mechanism for Nonconscious Activation of Conditioned Placebo and Nocebo Responses. *Cerebral cortex (New York, N.Y. : 1991), 25*(10), 3903-3910. doi:10.1093/cercor/bhu275

Kaptchuk, T. J., Kelley, J. M., Deykin, A., Wayne, P. M., Lasagna, L. C., Epstein, I. O., . . . Wechsler, M. E. (2008). Do "placebo responders" exist? *Contemp Clin Trials, 29*(4), 587-595. doi:10.1016/j.cct.2008.02.002

Levine, J. D., Gordon, N. C., Bornstein, J. C., & Fields, H. L. (1979). Role of pain in placebo analgesia. *Proc Natl Acad Sci U S A, 76*(7), 3528-3531. doi:10.1073/pnas.76.7.3528

Mokhtar, M., & Singh, P. (2020). Neuroanatomy, Periaqueductal Gray. *StatPearls [Internet]*.

Necka, E. A., Lee, I.-S., Kucyi, A., Cheng, J. C., Yu, Q., & Atlas, L. Y. (2019). Applications of dynamic functional connectivity to pain and its modulation. *PAIN Reports, 4*(4).

Olsen, M. F., Bjerre, E., Hansen, M. D., Tendal, B., Hilden, J., & Hróbjartsson, A. (2018). Minimum clinically important differences in chronic pain vary considerably by baseline pain and methodological factors: systematic review of empirical studies. *J Clin Epidemiol, 101*, 87-106.e102. doi:10.1016/j.jclinepi.2018.05.007

Ongür, D., An, X., & Price, J. L. (1998). Prefrontal cortical projections to the hypothalamus in macaque monkeys. *J Comp Neurol, 401*(4), 480-505.

Pagnini, F., Barbiani, D., Cavalera, C., Volpato, E., Grosso, F., Minazzi, G. A., . . . Phillips, D. (2023). Placebo and Nocebo Effects as Bayesian-Brain Phenomena: The Overlooked Role of Likelihood and Attention. *Perspect Psychol Sci*, 17456916221141383. doi:10.1177/17456916221141383

Petrovic, P., Kalso, E., Petersson, K. M., & Ingvar, M. (2002). Placebo and Opioid Analgesia-- Imaging a Shared Neuronal Network. *Science, 295*(5560), 1737. doi:10.1126/science.1067176

Peyron, R., Quesada, C., & Fauchon, C. (2019). Chapter 17 - Cingulate-mediated approaches to treating chronic pain. In B. A. Vogt (Ed.), *Handbook of Clinical Neurology* (Vol. 166, pp. 317-326): Elsevier.

Särkkä, S., Solin, A., Nummenmaa, A., Vehtari, A., Auranen, T., Vanni, S., & Lin, F.-H. (2012). Dynamic retrospective filtering of physiological noise in BOLD fMRI: DRIFTER. *NeuroImage, 60*, 1517-1527. doi:10.1016/j.neuroimage.2012.01.067

Schenk, L. A., & Colloca, L. (2019). The neural processes of acquiring placebo effects through observation. *NeuroImage*, 116510. doi:10.1016/j.neuroimage.2019.116510

Schmid, J., Langhorst, J., Gaß, F., Theysohn, N., Benson, S., Engler, H., . . . Elsenbruch, S. (2015). Placebo analgesia in patients with functional and organic abdominal pain: a fMRI study in IBS, UC and healthy volunteers. *Gut, 64*(3), 418-427. doi:10.1136/gutjnl-2013-306648

Scott, D. J., Stohler, C. S., Egnatuk, C. M., Wang, H., Koeppe, R. A., & Zubieta, J. K. (2008). Placebo and nocebo effects are defined by opposite opioid and dopaminergic responses. *Arch Gen Psychiatry, 65*(2), 220-231. doi:10.1001/archgenpsychiatry.2007.34

Stroman, P. W., Ioachim, G., Powers, J. M., Staud, R., & Pukall, C. (2018). Pain processing in the human brainstem and spinal cord before, during, and after the application of noxious heat stimuli. *Pain, 159*(10), 2012-2020. doi:10.1097/j.pain.0000000000001302

Vachon-Presseau, E., Berger, S. E., Abdullah, T. B., Huang, L., Cecchi, G. A., Griffith, J. W., . . . Apkarian, A. V. (2018). Brain and psychological determinants of placebo pill response in chronic pain patients. *Nat Commun, 9*(1), 3397. doi:10.1038/s41467-018-05859-1

Wager, T. D., Davidson, M. L., Hughes, B. L., Lindquist, M. A., & Ochsner, K. N. (2008). Prefrontal-subcortical pathways mediating successful emotion regulation. *Neuron, 59*(6), 1037-1050. doi:10.1016/j.neuron.2008.09.006

Wager, T. D., Rilling, J. K., Smith, E. E., Sokolik, A., Casey, K. L., Davidson, R. J., . . . Cohen, J. D. (2004). Placebo-induced changes in FMRI in the anticipation and experience of pain. *Science, 303*(5661), 1162-1167. doi:10.1126/science.1093065

Wager, T. D., Scott, D. J., & Zubieta, J. K. (2007). Placebo effects on human mu-opioid activity during pain. *Proc Natl Acad Sci U S A, 104*(26), 11056-11061. doi:10.1073/pnas.0702413104

Wang, W., Schuette, P. J., Nagai, J., Tobias, B. C., Cuccovia, V. R. F. M., Ji, S., . . . Adhikari, A. (2021). Coordination of escape and spatial navigation circuits orchestrates versatile flight from threats. *Neuron, 109*(11), 1848-1860 e1848. doi:10.1016/j.neuron.2021.03.033

Youssef, A. M., Macefield, V. G., & Henderson, L. A. (2016). Pain inhibits pain; human brainstem mechanisms. *NeuroImage, 124*(Pt A), 54-62. doi:10.1016/j.neuroimage.2015.08.060

Zubieta, J. K., Bueller, J. A., Jackson, L. R., Scott, D. J., Xu, Y., Koeppe, R. A., . . . Stohler, C. S. (2005). Placebo effects mediated by endogenous opioid activity on mu-opioid receptors. *J Neurosci, 25*(34), 7754-7762. doi:10.1523/jneurosci.0439-05.2005

Reviewers' comments:

Reviewer #1 (Remarks to the Author):

Stimulus-independent and stimulus-dependent neural networks underpin placebo responsiveness in humans.

The authors provide a very thorough revision of the manuscript addressing many of the reviewers' points raised in the previous review step.Unfortunately, the authors' clarification of the experimental and methodological procedures reveals that the main conclusions made by the authors are not properly backed up by the statistical analyses.

One such example is the arbitrary distinction into placebo responders and non-responders, rather than using the placebo response as a continuous variable. Dichotomizing data is associated with substantial loss of statistical power that can lead to biased results. See for instance:
https://onlinelibrary.wiley.com/doi/10.1002/pmrj.12228 or
https://pubmed.ncbi.nlm.nih.gov/11928888/

In their response the authors refer to previous publications that were based on such approach. However, the use of an inadequate approach in the past is not increase its validity to date. The authors also don't provide any information on the actual distribution of the placebo response in their sample. Does a histogram of the plotted placebo response indeed support two distinct populations? (e.g. two separate peaks?).
The authors explanation that baseline variations in pain perception would be better reflected in the dichotomous approach is not convincing. If the authors would want to capture that variance in their model they could add that variable. Further, additional information on pain ratings of the control conditions in the two groups does not support the idea of systematic differences. (see page 5 of the revised ms).

A second example of the methodological limitations is the statistical approach to test for PAG connectivity in placebo responders, as compared to non-responders. The authors report a significant main effect of placebo in the responder group, and the absence of such effect in the non-responder group, as revealed by T-Tests between conditions, but not between groups. While, as said above, this whole distinction into responders and non-responders is not ideal in the first place, (i) simply comparing a significant with a non-significant finding is statistically flawed and (ii) not finding a difference in one of the groups could be entirely due to a lack of statistical power. Connectivity patterns should be compared directly e.g. in terms of an interaction between group and condition.
There are more methodological aspects that warrant clarification, e.g. why no motor regressor was included in the analysis if participants provided continuous ratings of their pain perception, how were pain ratings between stimuli accounted for etc.

But in my view the first two points are that fundamental for the validity of the analyses that these should be addressed first, by showing that the key results hold, with an adequate statistical approach.

Reviewer #2 (Remarks to the Author):

The mns is well-revised. The authors addressed all suggestions except the consideration of placebo responders.

Dear Dr Welberg,

We thank the reviewer for their consideration of our manuscript and have made further changes to align with their final concern. We would however first like to discuss several points raised by the reviewer which we personally do not find accurately reflect the manuscript.

1) First, the distinction of responder and non-responder groups was not made arbitrarily, but rather by a well-supported, well-documented method for delineating subpopulations of participants which either show a significant or non-significant response to an experimental intervention. The two standard deviation band method is entirely distinct to previous methods such as arbitrary VAS changes which do vary within the literature, and we would agree are inferior to the present method employed. Indeed, our method revealed two distinct populations of data which align with our responder and non-responder groups. We have now generated a distribution plot of pain change data using 9 separate Bins of VAS change data in 5-point intervals to visualize the presence of two distinct populations as suggested by the reviewer, which can be found in Supplementary figure 1 (reproduced below). This plot is also now referenced within text on page 5 of the revised manuscript.

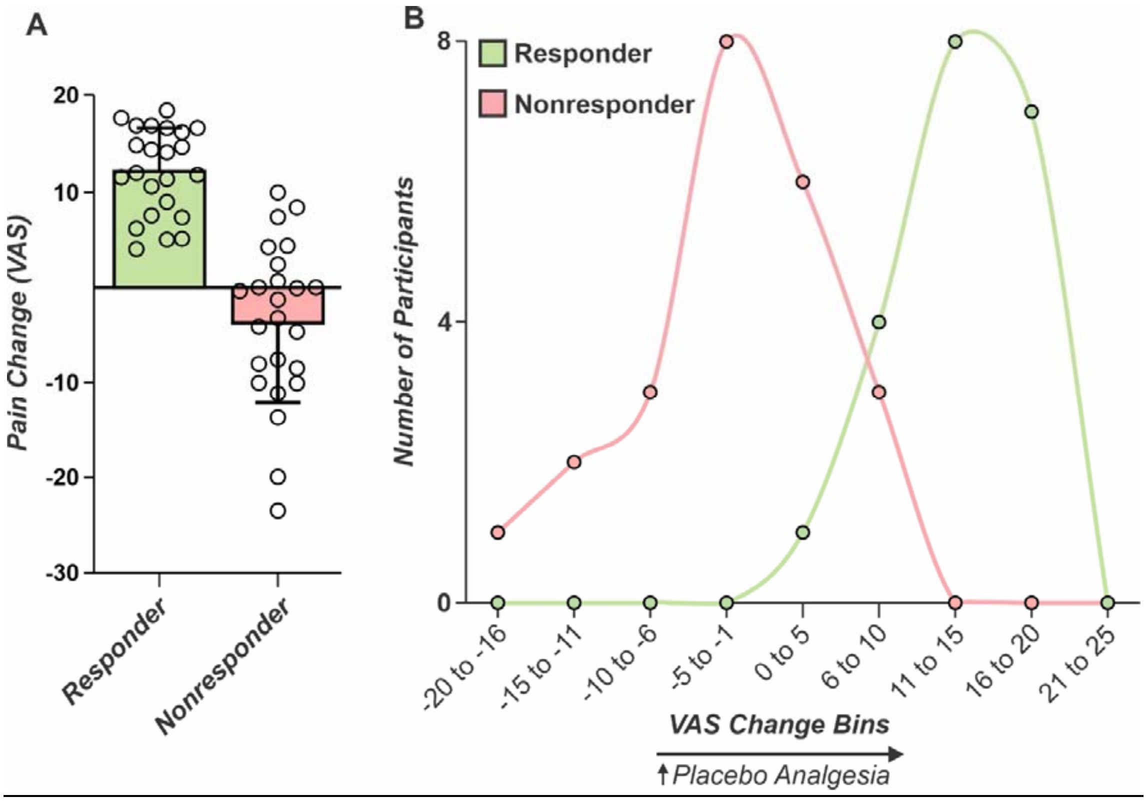

Figure 1. Individual participant pain change data and placebo analgesia distribution. A) Placebo responsiveness. Based on the two standard deviation band method, placebo responders and non-responders were delineated and changes in visual analogue scale (VAS)

values determined. More positive values indicate a greater placebo response when both control vaseline- and placebo lidocaine-sites received identical intensity stimuli during the test phase. **B) Distinct group-level difference in placebo analgesia distribution.** Evenly spaced bins covering the extent of VAS changes in all participants were generated and a count of participants satisfying the criteria of these bins in both placebo responder (green curve, n=23) and nonresponder (pink curve, n=24) groups were determined. More positive values indicate a greater placebo analgesia response.

2) Moreover, we find the issue of statistical power intriguing given we performed an apriori power analysis on a manuscript which included two groups: one which received naloxone (i.e. directly inhibited placebo response), and one which did not (i.e. experienced a placebo response) (Eippert et al. 2009). These two groups are reflective of a responder and non-responder group, and we determined a total sample size of 40 participants would be necessary to detect similar effect sizes with satisfactory power. Our sample of 47 participants, with a response rate exceeding that commonly observed within the literature which do perform responder:non-responder dichotomization we believe satisfies any concerns over reaching adequate statistical power without needing to perform correlative statistics.

Whilst the reviewer provides examples where dichotomizing a continuous variable does in fact lead to statistically misleading results, a similar argument can be drawn when correlative statistics are performed on data which contains non independent samples https://journals.sagepub.com/doi/10.1111/j.1745-6924.2009.01127.x. Not only can performing tests in this manner lead to a failure to detect real effects, but indeed the inverse, where r values can be *inflated* with a lack of statistical power. In line with our supplementary figure, elected methodology for performing dichotomization (i.e. not a median split or arbitrary threshold) and results from our power analysis, we suggest that dichotomizing our sample into a responder and non-responder group was not only valid, but the ideal method of analysis for investigating discrete changes in coupling related to significant placebo analgesia responses.

The seminal issue with performing correlative statistics on a phenomenon like placebo analgesia is that a voxel-by-voxel analysis (such as the connectivity analyses performed within

this manuscript) would yield clusters where connectivity change scales even in those whose pain did not change or *increased* when a placebo substance was administered. The fundamental flaw here is that these participants have *failed* to mount a pain modulatory phenomenon, and so considering them in the same correlation analysis as those that do successfully mount this phenomenon would hinder any investigation into the most important circuits involved in significant pain modulation. Indeed, our results show that a collection of top-down signals from the hypothalamus, rACC, and NAc are most integral in the manifestation of these responses, and that group-level differences do exist in those that produce the placebo analgesia phenomenon compared to those that do not.

In line with the statistical approach, we have now performed an additional analysis comparing whether the mean change in connectivity between the PAG seed and each cluster in the PPI and WSC analyses demonstrate group and condition level differences. This was performed by calculating the mean change in coupling between the control Vaseline- and placebo lidocaine-treated sites, and then performing a 2 sample T-test between the responder and non-responder groups mean change in connectivity values. All identified clusters in both analyses except the contralateral S1 in the PPI analysis exceeded significance, which is in line with the reported values – as both responders and non-responders displayed similar significant change in stimulus dependent coupling between the PAG seed and S1. These values and explanation of test performed are now found within the methods section of the manuscript as well as in tables 1 and 2.

Page 24:
From resulting clusters, eigenvariates representing stimulus-independent connectivity with the PAG in each series were extracted from both placebo responder and non-responder contrast images and significance was determined in both groups using paired t-tests (Figure 2B,C). To determine whether these changes in connectivity were significantly difference between placebo responder and non-responder groups, post minus pre connectivity difference values were generated for each group, and two sample t-tests performed ($p<0.05$) (Table 1).

Page 25:
From resulting clusters, eigenvariates representing stimulus-dependent connectivity with the PAG in each series were extracted from both placebo responder and non-responder contrast images and significance was determined in both groups using paired t-tests (Figure 2E, F). To

determine whether these changes in connectivity were significantly difference between placebo responder and non-responder groups, post minus pre connectivity difference values were generated for each group, and two sample t-tests performed ($p<0.05$) (Table 2).

Table 1. Location, level of significance, and cluster size of regions altering in connectivity with the right lateral PAG specifically during stimulus application in the placebo responder group. The level of significance between conditions and groups is provided in the right most column. Co-ordinates are in Montreal Neurological Institute (MNI) space. Cluster sizes are derived from resliced 1mm isotropic image series. "ipsilateral" = right. *NAcc = nucleus accumbens; rACC = rostral anterior cingulate cortex; dACC = dorsal anterior cingulate cortex; MCC = mid cingulate cortex; SMA = supplementary motor area; S1 = primary somatosensory cortex.*

	**MNI coordinates**			**t-value**	**cluster size**	**PAG Stimulus-dependent connectivity change (mean ± SEM)**		**responder:non-responder connectivity change (p-value)**
	**X**	**Y**	**Z**			**control scan**	**lidocaine scan**	
**Psychophysiological Interaction (PPI)** ***PBO>control***								
Contralateral Anterior Insula	-37<br>-33	12<br>13	-7<br>11	3.64<br>3.65	72<br>31	-0.26±0.12<br>-0.23±0.13	0.40±0.14<br>0.40±0.11	0.03<br>0.04
Contralateral NAcc	-7	5	-5	4.27	64	-0.26±0.06	0.16±0.10	0.003
Ipsilateral rACC	12	36	20	3.84	82	-0.14±0.06	0.34±0.10	0.02
Ipsilateral dACC	6	15	37	3.71	271	-0.26±0.13	0.59±0.20	0.008
Ipsilateral MCC	4	-16	40	3.62	50	-0.36±0.09	0.32±0.20	0.01
Ipsilateral SMA	4	8	45	4.31	180	-0.47±0.17	0.34±0.19	0.03
Contralateral S1	-38	-34	46	4.10	367	-0.45±0.17	0.39±0.14	0.28

Table 2. Location, level of significance, and cluster size of regions altering in connectivity with the right lateral PAG across the entire scan timecourse in the placebo responder group. The level of significance between conditions and groups is provided in the right most column. Co-ordinates are in Montreal Neurological Institute (MNI) space. Cluster sizes are derived from resliced 1mm isotropic image series. "ipsilateral" = right. *rACC = rostral anterior cingulate cortex; dlPFC = dorsolateral prefrontal cortex; mPFC = medial prefrontal cortex; MeA = medial nucleus of the amygdala.*

	**MNI coordinates**					**PAG Whole scan connectivity change (mean ± SEM)**		**responder:non-responder connectivity change (p-value)**
	**X**	**Y**	**Z**	**t-value**	**cluster size**	**control scan**	**lidocaine scan**	
**Functional Connectivity (FC)** *PBO>Control*								
Ipsilateral rACC	3	40	17	5.10	311	-0.009±0.008	0.035±0.006	0.01
Contralateral dlPFC	-29	25	27	4.16	98	-0.007±0.007	0.025±0.008	0.004
PBO>Control								
Ipsilateral mPFC	11	60	-18	4.58	203	0.029±0.011	-0.016±0.007	0.007
Ipsilateral MeA	21	-2	-13	3.90	44	0.025±0.007	-0.008±0.008	0.006
Ipsilateral Posterior Hypothalamus	3	-5	-8	4.64	40	0.023±0.006	-0.007±0.006	0.04
Contralateral Posterior Hypothalamus	-1	-4	-7	5.63	83	0.020±0.005	-0.012±0.006	0.01

3) For the concern regarding pain ratings between stimuli, the noxious stimuli applied throughout this investigation were acute in nature, and participants were instructed to rate their pain as they experienced it. In no participant did we observe pain ratings above 0/100 outside of periods where noxious stimuli were applied, and as such do not believe these periods should be accounted for in the model (even if they were to be, they would not alter the model as they would be encoded as 0 regardless).

We hope the changes made to the manuscript account for the reviewer's final concerns.

Regards,

Luke Henderson

References:

Eippert F, Bingel U, Schoell ED, Yacubian J, Klinger R, Lorenz J, Büchel C. 2009. Activation of the Opioidergic Descending Pain Control System Underlies Placebo Analgesia. Neuron. 63:533-543.

REVIEWERS' COMMENTS:

Reviewer #1 (Remarks to the Author):

The authors have provided further analyses to substantiate their approach and support their final conclusions.
